# Alterations in resting‐state functional connectivity relate to psychopathology trajectories during emerging adolescence

**DOI:** 10.1002/jcv2.70135

**Published:** 2026-05-30

**Authors:** Jenna Jones Devine, Garrett R. Hosterman, Jolee Sloss, Adrienne L. Romer

**Affiliations:** ^1^ Department of Psychology Virginia Polytechnic Institute and State University Blacksburg Virginia USA

**Keywords:** adolescence, general psychopathology, longitudinal, *p*‐factor, resting‐state functional connectivity, transdiagnostic

## Abstract

**Background:**

A general psychopathology “*p*‐factor” captures shared variation across psychiatric disorder categories and is associated with dysfunctions in cognitive control. Alterations in resting‐state functional connectivity (RSFC) of cognitive and attentional networks have been associated concurrently with the *p*‐factor in youth samples. However, we do not yet know whether these RSFC alterations prospectively relate to change in the p‐factor over time during the transition to adolescence, when many forms of disorder onset or worsen.

**Methods:**

We examined whether baseline RSFC was prospectively related to the trajectory of *p*‐factor scores over three years in 9344 preadolescents (age 9–10 at baseline) from the Adolescent Brain Cognitive Development Adolescent Brain Cognitive DevelopmentSM (ABCD) study. Using longitudinal multilevel modeling, we tested whether baseline within‐ and between‐network RSFC of the default mode (DMN), frontoparietal (FPN), salience (SN), cingulo‐opercular (CON), and ventral and dorsal attention (VAN and DAN) networks were related to the intercept (between‐person differences) and slope (within‐person rate of change) of p‐factor scores over four ABCD study waves (3‐year timeframe).

**Results:**

There was a significant nonlinear, quadratic trajectory of p‐factor scores over wave. Lower within‐DMN and within‐DAN connectivity and greater DMN‐DAN, DMN‐CON, and VAN‐CON connectivity were associated with higher between‐person levels of p‐factor scores, which persisted over time. Greater VAN‐DAN and SN‐CON connectivity and lower DMN‐VAN connectivity were prospectively associated with steeper within‐person rates of quadratic change in p‐factor scores over time.

**Conclusion:**

These novel results identify specific alterations in RSFC within and between core networks involved in self‐referential processing, bottom‐up and town‐down attention, salience, and cognitive control that might contribute to general psychopathology development in early adolescence. Children with aberrant connectivity between the DMN and VAN, SN and CON, and VAN and DAN networks may be vulnerable to increases in general psychopathology during the transition to adolescence.

## Key Points

**What's known?**

- A general psychopathology factor, often called the ‘*p*‐factor’, captures comorbidity and severity of mental disorders across diagnostic categories.
- The p‐factor is associated with abnormal patterns of resting‐state functional connectivity (RSFC) in young people, but less is known about whether these abnormalities are risk markers for future general psychopathology development during adolescence.

**What's new?**

- We identified specific alterations in RSFC within and between networks supporting self‐focused thought, attention, and cognitive control that might contribute to general psychopathology development in early adolescence.

**What's relevant?**

- RSFC of networks involved in cognitive and attentional control may be markers of risk for increases in general psychopathology during the adolescent vulnerability period.
- Findings suggest that youth interventions that aim to strengthen cognitive and attentional control may be effective for addressing symptoms across diagnostic categories.

## INTRODUCTION

Current categorical classification systems of psychological disorders contain high levels of symptom heterogeneity and fail to fully address mental disorder comorbidity, which is estimated to be as high as 40% in young people (Merikangas et al., 2010; Segal et al., 2024). Increasing research has highlighted the need to study transdiagnostic, dimensional characteristics of psychopathology across traditional diagnostic categories. Factor‐analytic studies have identified a general factor of psychopathology, often called the “*p*‐factor”, which captures the shared variation of symptoms across categorical disorders, accounting for their comorbidity and severity (Caspi et al., 2014; Caspi & Moffitt, 2018). The *p*‐factor has been identified in many varied samples of children, adolescents, and adults (Caspi & Moffitt, 2018; Lahey et al., 2017), has replicable neurobiological and cognitive correlates (Hoy et al., 2023), and predicts serious adverse outcomes. For example, over and above specific mental disorder categories, higher childhood levels of the *p*‐factor have been associated with greater future mental disorder diagnoses, psychotropic medication prescriptions, and criminal convictions, and lower educational outcomes in adolescence and adulthood (Caspi et al., 2014; Pettersson et al., 2018; Sallis et al., 2019). Thus, studying early neurobiological and psychological markers of the p‐factor may be important for identifying youth at risk for developing comorbid psychopathology.

Dysfunctions in cognitive and attentional processing may be one such marker of the *p*‐factor. Specifically, poorer performance on cognitive tests has been associated concurrently with higher levels of the p‐factor in studies of children (Cardenas‐Iniguez et al., 2020; Huang‐Pollock et al., 2017; Martel et al., 2017), adolescents (Bloemen et al., 2018; Castellanos‐Ryan et al., 2016; Snyder et al., 2019; Wade et al., 2019; White et al., 2017), and adults (Caspi et al., 2014; Romer & Pizzagalli, 2022). Further, childhood cognitive function has been shown to prospectively predict future p‐factor scores in adolescence and adulthood (Caspi et al., 2014; Romer & Pizzagalli, 2021). Neuroimaging and genetics research also supports the hypothesis that early cognitive dysfunctions may contribute to general psychopathology. Youth twin studies have identified shared genetic influences on cognitive function and transdiagnostic psychopathology (Alnæs et al., 2018; Grotzinger et al., 2019; Harden et al., 2020). Structural and functional neural abnormalities in regions involved in cognitive and attentional processes also have been identified in transdiagnostic meta‐analyses (Goodkind et al., 2015; McTeague et al., 2016; Shanmugan et al., 2016) and in studies of the neural correlates of the p‐factor (Elliott et al., 2018; Moberget et al., 2019; Romer et al., 2018, 2021; Snyder et al., 2017). Thus, examining childhood alterations in neural networks that support cognitive and attentional processes may be important for identifying neurocognitive markers of general psychopathology.

Resting‐state functional connectivity (RSFC) is a powerful tool that can be used to examine the intrinsic organization of neural networks involved in cognitive and attentional functions. Broadly, RSFC captures temporal correlations of neural activation in anatomically distinct but functionally related regions of the brain, providing an architecture for understanding how large‐scale neural networks organize information and interact with each other when not engaged in a specific task (Fox et al., 2005; Glover, 2011; Woodward & Cascio, 2015). Converging work from structural, functional, behavioral, and lesion studies have identified unique resting‐state networks with distinct functions (Gordon et al., 2016). Functional connectivity of these networks can be measured both as patterns of connectivity within networks (i.e., co‐activation among the nodes within a network) as well as between networks (i.e., patterns of co‐activation between nodes across networks).

According to Menon's (2011) triple network model, aberrant patterns of connectivity within and between core cognitive networks, including the default mode (DMN), frontoparietal (FPN), and salience (SN) networks, may be associated with psychopathology broadly. Specifically, the DMN is involved in self‐referential thought (e.g., self‐reflection, mind wandering) and is active when the brain is “at rest” (Fox et al., 2005; Menon, 2011; Raichle, 2015), whereas the FPN is a top‐down control network involved in “cold” executive functions such as maintaining and manipulating memory, decision‐making, and executing goal‐directed behaviors (Marek & Dosenbach, 2018; Menon & D’Esposito, 2022). The SN is responsible for detecting, filtering, and integrating relevant information and is thought to serve as a “switch” between the DMN and FPN, such that when an external stimulus is important enough to warrant attention, the SN is involved in deactivating the DMN and engaging the FPN (Menon, 2011; Menon & D’Esposito, 2022; Seeley, 2019). Menon's (2011) triple network model suggests that dysfunctions in the appropriate engagement or disengagement of these three networks in the context of stimulus‐driven cognitive and affective information may contribute to symptoms across a range of disorder categories.

Results from transdiagnostic RSFC meta‐analyses and studies of the p‐factor are largely consistent with Menon's (2011) triple network model. For example, a RSFC meta‐analysis found lower DMN‐ventral SN and SN‐FPN connectivity and greater DMN‐FPN and DMN‐dorsal SN connectivity across eight categorical disorders compared to healthy controls (Sha et al., 2019). Decreased segregation of the DMN with the FPN and SN also was shared across transdiagnostic dimensions of psychopathology in the Philadelphia Neurodevelopmental Cohort of youth ages 8–23 (Xia et al., 2018). Hyperconnectivity between higher‐order visual and DMN and FPN networks were associated concurrently with higher p‐factor scores in the Duke Neurogenetics Study of young adults (ages 18–22) (Elliott et al., 2018). In the Adolescent Brain and Cognitive Development (ABCD) study of preadolescents (ages 9–10 at baseline), higher p‐factor scores were associated concurrently with lower within‐DMN and greater DMN‐FPN connectivity (Karcher et al., 2021; Lees et al., 2021; Sripada et al., 2021). These studies also identified alterations in the connectivity of the ventral and dorsal attention (VAN and DAN) and cingulo‐opercular (CON) networks involved in bottom‐up and top‐down attention and cognitive control, respectively. Specifically, lower within‐DAN and greater DMN‐CON, DMN‐DAN, and FPN‐VAN connectivity was associated concurrently with higher p‐factor scores in the ABCD study (Karcher et al., 2021; Lees et al., 2021; Sripada et al., 2021). This research suggests that RSFC alterations within and between these six cognitive and attentional networks (DMN, FPN, SN, VAN, DAN, CON) may be associated with general psychopathology.

However, much of this research has been cross‐sectional. We do not yet know whether relations between aberrant connectivity of these cognitive and attentional RSFC networks and the *p*‐factor will persist over time or prospectively relate to rates of change in the *p*‐factor. Conducting such prospective longitudinal RSFC research may be especially important during the transition to adolescence, when extensive neurodevelopmental changes occur (Gu et al., 2015; Morgan et al., 2018; Stevens, 2009) and many forms of psychiatric disorder onset or worsen (Solmi et al., 2022). Identifying childhood RSFC alterations that prospectively relate to levels and/or rates of change in general psychopathology has implications for early identification of children who may be vulnerable to developing comorbid and severe forms of disorder in adolescence.

Thus, in the present study, we examined whether RSFC alterations within and between core cognitive and attentional networks (DMN, FPN, SN, VAN, DAN, CON) are prospectively related to levels of and rates of change in p‐factor scores during the transition to adolescence. To do this, we employed four waves of clinical data and the first wave of RSFC data from the ABCD study. We calculated unstandardized factor scores from previously identified higher‐order confirmatory factor models of the structure of psychopathology using the same clinical scales at each wave (Romer et al., 2023; Romer & Pizzagalli, 2021). We then performed longitudinal multilevel modeling to examine whether within‐ and between‐network RSFC at baseline prospectively related to the intercept (between‐person differences) and slope (within‐person rate of change) of p‐factor scores over four study waves (3‐year time period). Based on prior cross‐sectional RSFC research in the ABCD study (Karcher et al., 2021; Lees et al., 2021; Sripada et al., 2021), we hypothesized that lower connectivity within‐DMN and within‐DAN networks and greater connectivity between FPN, SN, VAN, DAN, and CON networks would be associated with higher between‐person p‐factor scores. We considered analyses examining whether RSFC relates to rates of change in p‐factor scores over time to be exploratory, as no known studies have investigated this question. Finally, we also conducted exploratory analyses of RSFC networks involved in more basic, unimodal processes (i.e., visual, auditory, cingulo‐parietal, retrospenial temporal, sensorimotor mouth and hand, and “none” networks) to determine whether RSFC of non‐cognitive networks may also prospectively relate to p‐factor scores during the transition to adolescence.

## METHODS AND MATERIALS

### Participants

Data from the ABCD study were accessed from the National Institute of Mental Health Data Archive (NDA) 5.1 release (DOI: 10.15154/z563‐zd24). The ABCD sample consists of 11,875 youth who participated in a major collaboration across 21 sites nationwide. Complete recruitment details can be found elsewhere (Garavan et al., 2018). Exclusion criteria included: not being fluent in English; having a parent not fluent in English or Spanish; major medical or neurological conditions; gestational age <28 weeks or birthweight <1200 g; contraindications to MRI scanning; history of traumatic brain injury; current schizophrenia diagnosis; moderate/severe autism spectrum disorder; intellectual disability; or alcohol/substance use disorder. Institutional review board approval was obtained for each site before data collection. All parents provided written informed consent and children provided assent. Participants were excluded from the present study analyses for the following reasons: missing demographic information; complete nonresponse on clinical data; participated at a study site with less than 40 participants; did not pass MRI quality assurance measures (see Supporting Information S1; Figure S1 and Appendix S1 for details) (baseline Wave 1 *n* = 9344; 1‐year follow‐up Wave 2 *n* = 8840; 2‐year follow‐up Wave 3 *n* = 8618; 3‐year follow‐up Wave 4 *n* = 8034).

### Psychopathology

Psychopathology was assessed at each wave using the Child Behavior Checklist (CBCL), a 119‐item form measuring child behavior and emotion (Achenbach, 2011). Parents rate their child's emotions and behaviors on a scale of 0 (“Not True [as far as you know]”), 1 (“Somewhat or Sometimes True”), or 2 (“Very True or Often True”).

### Covariates

Sex assigned at birth and baseline age (in months) for each child was reported by their parent/guardian. MRI Scanner type was dummy‐coded as Prisma, Discovery, Achieva, and Ingenia variables (Prisma Fit = reference group). Motion in the scanner was captured by mean framewise displacement (FD; measured in millimeters; variable “rsfmri_meanmotion”). Current (past 2 weeks) medication use also was reported by parents/guardians, which was included as a covariate in sensitivity analyses.

### Imaging acquisition and processing

A detailed description of full imaging acquisition and processing procedures is available elsewhere (Casey et al., 2018; Hagler et al., 2019) and in the Supporting Information S1; Appendix S1. Briefly, brain data were collected on 3T scanners (Siemens Prisma and Prisma Fit, General Electric MR 750, Philips Achieva dStream and Ingenia). Participants completed two resting‐state functional MRI scans. Following image acquisition, the ABCD Data Analysis and Informatics Resource Center (DAIRC) inspected the data for protocol adherence and completeness and performed standardized processing procedures, including head motion and B0 distortion corrections. Following image preprocessing, data were further processed by the DAIRC prior to analyses. Initial frames were removed, voxel time series normalized, regressed to remove quadratic trends, signals correlated with motion removed, and mean time courses for each region‐of‐interest (ROI) calculated.

### Resting‐state functional connectivity (RSFC)

Preprocessed time courses for each individual were sampled onto the cortical surface. Functionally defined parcellations (Gordon et al., 2016) then were resampled from atlas‐space to individual subject‐space. For each of the 13 Gordon networks, within‐network RSFC was calculated by averaging Fisher‐transformed pairwise correlations of all ROIs comprising a specific network. Between‐network RSFC was calculated by averaging all unique Fisher‐transformed pairwise correlations of all ROIs in the first network with all ROIs in the second network. To further reduce residual effects of head motion, timepoints with a FD greater than 0.2 mm were excluded from these correlation calculations. Finally, correlation values were averaged across the four resting‐state scans (weighted by number of remaining frames after censoring for motion) to calculate within‐ and between‐network RSFC.

### Statistical analyses

#### Confirmatory factor analysis (CFA)

Previously, higher‐order factor models of CBCL item‐level data were fit at each of the first three study waves and demonstrated longitudinal measurement invariance (for more details, see Romer et al., 2023, Romer & Pizzagalli, 2021 and Supporting Information S1; Appendix S2 and Appendix S3). The higher‐order model identified five lower‐order factors (externalizing, internalizing, neurodevelopmental, somatization, and detachment) and a higher‐order p‐factor, consistent with the HiTOP framework (Kotov et al., 2017; Michelini et al., 2019). In the present study, we additionally fit this higher‐order model to the wave 4 CBCL item‐level data, which provided an adequate fit and was found to be scalar invariant over all four waves (i.e., equivalent factor loadings and response category thresholds) (see Supporting Information S1; Tables S1–S3), supporting the examination of within‐person change in the factor scores over time. Thus, as in our prior work (Romer et al., 2023), we calculated p‐factor scores at each wave by multiplying each CBCL item by its unstandardized factor loading from the baseline model and then summing the weighted items, with higher scores reflecting greater comorbidity and severity of symptoms.

#### Longitudinal multilevel modeling

We conducted two‐level growth models to test associations between baseline RSFC and p‐factor score trajectories over 3 years. Level 1 accounted for the within‐subject trajectory of p‐factor scores and Level 2 accounted for between‐subject differences in p‐factor scores. We included subject‐specific random intercepts and slopes for time. Time was coded as wave number from 0 (baseline wave 1) to 3 (3‐year follow‐up/wave 4). Analyses were conducted in *R* version 4.5.0 (http://www.r‐project.org/) using the lme4 package. First, we determined the best‐fitting functional form of the trajectory of p‐factor scores over wave by testing and comparing the fit of unconditional linear and nonlinear growth models. Second, we tested whether within‐ and between‐network RSFC among the six cognitive and attentional networks (DMN, FPN, SN, VAN, DAN, CON) were related to the intercept (between‐person differences) and slope (within‐person rate of change) of p‐factor scores over four ABCD study waves (3‐year timeframe), for a total of 21 growth models tested. Additionally, we conducted exploratory analyses of within‐ and between‐network RSFC of all remaining Gordon networks (auditory, cingulo‐parietal, retrosplenial temporal, sensorimotor hand, sensorimotor mouth, visual, and “none” networks) and their connectivity with the six cognitive and attentional networks, for a total of 70 growth models tested. Baseline within‐ and between‐network RSFC measures were grand‐mean‐centered and included as time‐invariant covariates (TICs). Sex assigned at birth, age (in months), mean FD, and MRI scanner model (dummy‐coded) at baseline were included as Level 2 TICs of no interest. Additionally, to control for nesting within study site and family, we included random intercepts for site and family unit in the models.

The conditional growth models were conducted using the following fixed effects formula: p‐factor scores = *β*_1_*wave + *β*_2_*wave^2^ + *β*_3_*age at baseline + *β*_4_*sex + *β*_5_*MRI scanner dummies + *β*_6_*mean FD + *β*_7_*RSFC network + *β*_8_*RSFC network × wave + *β*_9_*RSFC network × wave^2^. Missing data was assumed to be missing‐at‐random and the likelihood of the models based on the observed data was sufficient for inference on the associations of interest. Thus, all participants with at least one observation were included in the growth models. We corrected for multiple comparisons by using a false discovery rate (FDR) procedure (Benjamini & Hochberg, 1995) (*q* < 0.05) for the 42 hypothesis‐driven tests in one set (21 growth models including intercept and slope effects) and for the 140 exploratory tests in a second set (70 growth models including intercept and slope effects). Analysis code is available at https://github.com/Ageyr13/ABCD_RSFC_MLM.git. Associations between RSFC networks and intercepts would indicate that baseline RSFC is associated with initial levels of p‐factor scores, whereas associations with slope would indicate that within‐person p‐factor trajectories differ as a function of baseline RSFC. If significant relations between RSFC and intercepts were identified, but not slopes, we tested whether these associations remained significant at waves 2, 3, and 4 to determine the persistence of these relations over wave.

### Sensitivity analyses

To test the robustness of our findings, we conducted the following sensitivity analyses: (1) excluded participants with mean FD > 0.2 mm to further control for the effects of head motion; (2) included psychotropic medication use as an additional TIC that may influence both RSFC and psychopathology; and (3) excluded participants with completely missing follow‐up clinical data.

## RESULTS

### Descriptive statistics

Descriptive statistics and missingness information for all study variables are included in Table 1 (see Supporting Information S1; Table S4 for bivariate correlations). Of the 9344 participants included at baseline, 1601 had missing CBCL data at one or more of the follow‐up waves. Of those participants with missing follow‐up data, 270 participants were missing at all three follow‐up waves. Baseline differences in all study variables between participants with any missing follow‐up data (*n* = 1601) versus those with no missing follow‐up data (*n* = 7743) were tested. The sample with no missing follow‐up data had significantly lower mean FD, lower baseline p‐factor scores, lower DMN‐DAN, SN‐DAN, and VAN‐DAN connectivity and higher CON‐CON connectivity compared to those with missing data. Given that there are differences between those with and without missing data on baseline p‐factor scores, we conducted sensitivity analyses by removing the 270 participants with completely missing CBCL follow‐up data.

**TABLE 1:** Descriptive statistics of study variables and comparisons of participants with and without missing follow‐up data.

	Baseline full sample				Non‐missing sample		Missing sample		*X* ^2^/*t*	*p*‐value
	*n*	Min	Max	Mean (SD) or %	*n*	Mean (SD) or %	*n*	Mean (SD) or %		
Demographics and covariates										
Age (months)	9348	107	133	119.25 (7.52)	7743	119.28 (7.55)	1605	119.10 (7.39)	0.88	0.377
Sex (% female)	9348			49.74	7743	49.53	1605	50.77	0.783	0.376
Non‐Hispanic white (%)	9346			53.60	7743	56.79	1603	38.18	2860.1	**<0.001**
Black (%)	9346			13.72	7743	11.66	1603	23.64	216.15	**<0.001**
Asian (%)	9346			2.00	7743	1.95	1603	2.18	72.344	**<0.001**
Hispanic (%)	9346			20.23	7743	19.20	1603	25.20	623.2	**<0.001**
Other (%)	9346			10.46	7743	10.40	1603	10.79	408.41	**<0.001**
Average FD	9336	0.02	2.49	0.23 (0.22)	7732	0.23 (0.22)	1604	0.26 (0.23)	4.68	**<0.001**
No medication use (%)	9335			53.50	7736	52.51	1599	58.29	4.256	**<0.001**
*P*‐factor scores										
P1	9344	0	57.14	7.03 (7.46)	7743	6.82 (7.23)	1601	8.03 (8.42)	5.37	**<0.001**
P2	8840	0	50.63	6.75 (7.16)						
P3	8618	0	53.65	6.43 (7.04)						
P4	8034	0	54.02	6.46 (7.04)						

	**All waves**				**Non‐missing sample**		**Missing sample**		***X*** ^**2**^ **/*t***	*p* **‐value**
	***n***	**Min**	**Max**	**Mean (SD) or %**	***N***	**Mean (SD) or %**	***n***	**Mean (SD) or %**		
Resting‐state functional connectivity										
DMN‐DMN	9336	0.04	0.49	0.24 (0.06)	7732	0.24 (0.06)	1604	0.24 (0.06)	1.25	0.211
DMN‐FPN	9336	−0.13	0.23	0.05 (0.04)	7732	0.05 (0.04)	1604	0.05 (0.04)	1.46	0.144
DMN‐SN	9336	−0.21	0.33	0.07 (0.06)	7732	0.08 (0.06)	1604	0.07 (0.06)	2.28	0.023
DMN‐VAN	9335	−0.09	0.30	0.09 (0.05)	7731	0.09 (0.05)	1604	0.09 (0.05)	2.97	0.003
DMN‐DAN	9336	−0.37	0.06	−0.13 (0.05)	7732	−0.13 (0.05)	1604	−0.13 (0.06)	4.55	**<0.001**
DMN‐CON	9336	−0.13	0.23	0.05 (0.04)	7729	−0.11 (0.05)	1604	−0.11 (0.06)	1.91	0.056
FPN‐FPN	9336	0.04	0.48	0.21 (0.06)	7732	0.21 (0.06)	1604	0.22 (0.06)	0.97	0.334
FPN‐SN	9336	−0.17	0.38	0.09 (0.06)	7732	0.09 (0.06)	1604	0.09 (0.06)	1.50	0.134
FPN‐VAN	9335	−0.15	0.18	0.02 (0.04)	731	0.02 (0.04)	1604	0.02 (0.04)	0.94	0.348
FPN‐DAN	9336	−0.11	0.25	0.06 (0.04)	7732	0.06 (0.04)	1604	0.06 (0.04)	2.16	0.031
FPN‐CON	9333	−0.22	0.16	−0.01 (0.05)	7729	−0.01 (0.04)	1604	−0.01 (0.05)	2.85	0.004
SN‐SN	9336	−0.05	0.85	0.40 (0.12)	7732	0.40 (0.12)	1604	0.39 (0.12)	0.87	0.383
SN‐VAN	9335	−0.19	0.36	0.09 (0.07)	7731	0.09 (0.07)	1604	0.08 (0.06)	2.53	0.011
SN‐DAN	9336	−0.31	0.18	−0.04 (0.06)	7732	−0.04 (0.06)	1604	−0.04 (0.06)	4.78	**<0.001**
SN‐CON	9333	−0.12	0.46	0.13 (0.07)	7729	0.12 (0.07)	1604	0.13 (0.07)	1.07	0.283
VAN‐VAN	9335	0.04	0.51	0.24 (0.06)	7731	0.24 (0.06)	1604	0.23 (0.06)	1.36	0.175
VAN‐DAN	9335	−0.32	0.08	−0.08 (0.05)	7731	−0.08 (0.05)	1604	−0.08 (0.05)	4.81	**<0.001**
VAN‐CON	9333	−0.17	0.27	0.03 (0.05)	7728	0.02 (0.05)	1604	0.03 (0.05)	0.81	0.417
DAN‐DAN	9336	0.05	0.55	0.27 (0.07)	7732	0.28 (0.07)	1604	0.27 (0.07)	2.71	0.007
DAN‐CON	9333	−0.06	0.33	0.09 (0.05)	7729	0.09 (0.05)	1604	0.09 (0.05)	2.57	0.010
CON‐CON	9333	0.09	0.60	0.31 (0.07)	7729	0.31 (0.07)	1604	0.30 (0.07)	3.75	**<0.001**

*Note*: Comparisons were made by chi‐square tests for categorical variables and independent samples *t*‐tests for continuous variables. *p*‐values are unadjusted; *p*‐values that survived FDR correction for the 30 tests (*q* < 0.05) are indicated in bold. Slight variations in sample size reflect missing data in some variables.

Abbreviations: 1, baseline Wave 1; 2, 1‐year follow‐up Wave 2; 3, 2‐year follow‐up Wave 3; 4, 3‐year follow‐up Wave 4; CON, cingulo‐opercular network; DAN, dorsal attention network; DMN, default mode network; FPN, frontoparietal network; P, general psychopathology factor scores; SN, salience network; VAN, ventral attention network.

### Unconditional linear and non‐linear growth models

Likelihood ratio testing was employed to compare the fit of linear and non‐linear (quadratic) unconditional growth models of the trajectory of p‐factor scores. We found that the quadratic model provided a better fit to the data than the linear model (*X* ^2^ (4) = 36.353, *p* < 0.001), which was then employed in all subsequent conditional growth models. Random effects indicated that there were significant intraindividual (i.e., within‐subject) and interindividual (i.e., between‐subject) variability in the linear and quadratic intercepts and slopes (Table 2). There was more interindividual variability in the intercepts than intraindividual variability in the slopes, with minimal variability in the slopes of p‐factor scores over wave. Fixed effects indicated that p‐factor scores initially decreased from baseline to wave 3 (ages 9–12) and then began to increase from waves 3 to 4 (ages 12–13), with an overall increasing quadratic trend over time (*β* = 0.083, *p* < 0.001). The intra‐subject correlation for p‐factor scores was 0.781.

**TABLE 2:** Unconditional linear and quadratic growth models of P‐factor scores over four waves.

	*β*	SE	95% CI
Fixed effects			
Intercept	7.068***	0.199	[6.654, 7.482]
Wave	−0.416***	0.060	[−0.535, −0.298]
Wave^2^	0.083***	0.019	[0.046, 0.120]
	**Variance**	**SD**	**95% CI**
Random effects			
Level 2 intercept	19.322	4.396	[4.396, 4.400]
Level 2 linear slope	3.943	1.986	[1.626, 2.290]
Level 2 intercept‐linear slope correlation	−0.410		[−0.497, −0.327]
Level 2 quadratic slope	0.202	0.450	[0.269, 0.577]
Level 2 intercept‐quadratic slope correlation	0.060		[−0.104, −0.197]
Level 1 residual	11.450	3.384	[3.381, 3.424]

*Note*: Unstandardized estimates are shown. Level 1 Residual = intraindividual variability (within‐subject repeated measures); Level 2 = interindividual variability (between‐subject). ***p < 0.001. Wave^2^ refers to the quadratic fixed effect. Wave refers to the linear fixed effect.

Abbreviations: CI, confidence interval; SE, standard error.

### Within‐ and between‐network RSFC relations with *p*‐factor trajectories

**FIGURE 1:**
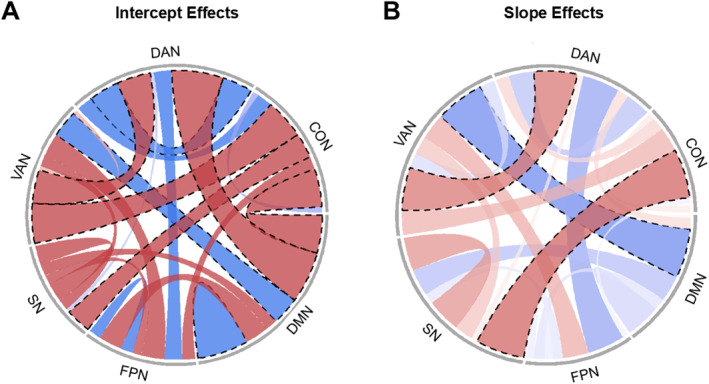
Intercept and Slope Effects of Altered Connectivity within and between Resting‐State Functional Connectivity Networks. (A) illustrates the intercept effect and (B) illustrates the quadratic slope effect of the association between altered baseline RSFC and p‐factor scores over time, over and above age, sex, scanner type, and in‐scanner motion. Link width and color are scaled by strength of association (wider, darker links = stronger effects). Intercept effects ranged from *β* = −0.032–0.055. Slope effects ranged from *β* = −0.008 to 0.008. Negative associations are shown in blue and positive associations are shown in red. Effects that were significant after false discovery rate (FDR) correction (*q* < 0.05) are outlined in black dashed lines. CON, cingulo‐opercular network; DAN, dorsal attention network; DMN, default mode network; FPN, frontoparietal network; SN, salience network; VAN, ventral attention network.

Associations between RSFC networks and intercepts and slopes (quadratic and linear) of *p*‐factor scores over wave are shown in Table 3 and Figure 1. As the unconditional quadratic model provided a better fit to the *p*‐factor score data over time than the linear model, we interpret the quadratic, rather than the linear, slopes below.

**TABLE 3:** Within‐ and between‐network resting‐state functional connectivity relations with intercepts and slopes of *p*‐factor scores over wave.

Resting‐state functional connectivity networks	Intercept (main effect)		Quadratic Slope (Interaction Effect)		Linear Slope (Interaction Effect)	
	*β*	95% CI	*Β*	95% CI	*β*	95% CI
DMN‐DMN	−0.027*	[−0.049, −0.006]	−0.003	[−0.008, 0.002]	0.014	[−0.003, 0.030]
DMN‐FPN	0.025*	[0.005, 0.045]	−0.002	[−0.007, 0.003]	0.002	[−0.015, 0.018]
DMN‐SN	0.013	[−0.007, 0.033]	−0.004	[−0.010, 0.001]	0.012	[−0.004, 0.029]
DMN‐VAN	−0.030**	[−0.050, −0.009]	−0.008**	[−0.013, −0.003]	0.029***	[0.013, 0.045]
DMN‐DAN	0.055***	[0.034, 0.076]	0.002	[−0.003, 0.007]	−0.015	[−0.031, 0.001]
DMN‐CON	0.041***	[0.020, 0.062]	0.000	[−0.005, 0.006]	−0.006	[−0.023, 0.010]
FPN‐FPN	−0.013	[−0.033, 0.008]	−0.001	[−0.006, 0.004]	0.001	[−0.016, 0.017]
FPN‐SN	0.010	[−0.011, 0.030]	−0.002	[−0.007, 0.003]	−0.004	[−0.021, 0.012]
FPN‐VAN	0.025*	[0.005, 0.045]	0.004	[−0.001, 0.009]	−0.016	[−0.032, 0.001]
FPN‐DAN	−0.019	[−0.039, 0.001]	−0.006*	[−0.011, −0.001]	0.023**	[0.007, 0.039]
FPN‐CON	0.014	[−0.006, 0.034]	−0.001	[−0.006, 0.004]	−0.002	[−0.018, 0.015]
SN‐SN	0.021*	[0.000, 0.041]	0.006*	[0.000, 0.011]	−0.023**	[−0.039, −0.007]
SN‐VAN	0.012	[−0.009, 0.032]	0.003	[−0.002, 0.008]	−0.009	[−0.026, 0.007]
SN‐DAN	−0.004	[−0.024, 0.016]	0.001	[−0.004, 0.006]	−0.003	[−0.019, 0.014]
SN‐CON	0.027**	[0.007, 0.047]	0.008**	[0.003, 0.013]	−0.025**	[−0.042, −0.009]
VAN‐VAN	0.004	[−0.016, 0.025]	−0.002	[−0.008, 0.003]	0.005	[−0.011, 0.022]
VAN‐DAN	0.036***	[0.016, 0.057]	0.007**	[0.002, 0.012]	−0.026**	[−0.043, −0.01]
VAN‐CON	0.043***	[0.022, 0.063]	0.004	[−0.001, 0.009]	−0.014	[−0.031, 0.002]
DAN‐DAN	−0.032**	[−0.053, −0.012]	−0.004	[−0.009, 0.001]	0.017*	[0.001, 0.033]
DAN‐CON	−0.017	[−0.037, 0.003]	0.002	[−0.003, 0.007]	−0.004	[−0.02, 0.013]
CON‐CON	−0.006	[−0.027, 0.015]	0.001	[−0.004, 0.006]	0.002	[−0.014, 0.019]

*Note*: The intercept and quadratic slope effects are the outcomes of interest in the present analysis. Intercept effects can be interpreted as the main effect of baseline RSFC on between‐person differences in p‐factor scores. Quadratic slope effects can be interpreted as interactions between baseline RSFC and wave, over and above linear slope. Covariates age, sex, scanner (dummy‐coded) are not shown here. * unadjusted *p* < 0.05; ** unadjusted *p* < 0.01; *** unadjusted *p* < 0.001. Standardized estimates are shown. **Bolded** estimates survived FDR correction (*q* > 0.05) for the 63 tests.

Abbreviations: CI, confidence interval; CON, cingulo‐opercular network; DAN, dorsal attention network; DMN, default mode network; FPN, frontoparietal network; SN, salience network; VAN, ventral attention network.

Lower within‐DMN and within‐DAN connectivity and greater DMN‐DAN, DMN‐CON, and VAN‐CON RSFC at baseline were significantly associated with higher initial levels (intercepts) of but not rates of change (quadratic slopes) in p‐factor scores after FDR correction. However, these between‐person associations remained significant at waves 2, 3, and 4 (Supporting Information S1; Table S5), demonstrating their persistence over time. Exploratory analyses of the non‐cognitive RSFC networks revealed no significant associations with the intercept or slope of p‐factor scores over wave after FDR correction (Supporting Information S1; Table S6).

**FIGURE 2:**
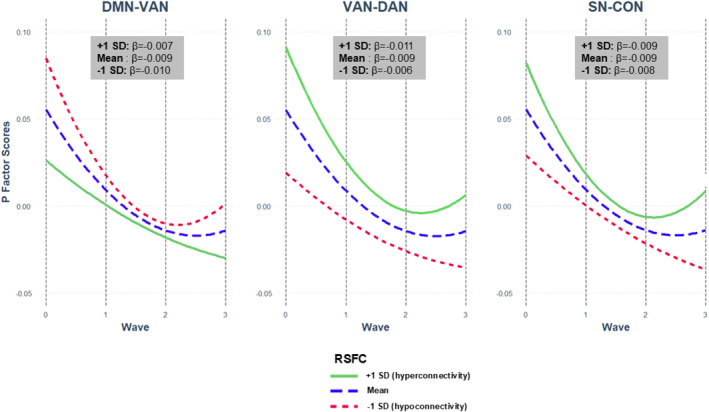
Baseline resting‐state functional connectivity (RSFC) Relations with the Rate of Change in *p*‐Factor Scores over Wave. The interaction between baseline RSFC and wave (slope) for *p*‐factor scores is illustrated. Estimated marginal mean analysis revealed that preadolescents with lower DMN‐VAN connectivity (1SD below mean) and higher VAN‐DAN and SN‐CON connectivity (1SD above mean) showed the steepest rates of quadratic change in p‐factor scores over time. All simple slopes are *p* < 0.001. 1SD above the mean represents “hyperconnectivity” and 1SD below the mean represents “hypoconnectivity.” CON, cingulo‐opercular network. DAN, dorsal attention network; DMN, default mode network; FPN, frontoparietal network; SD, standard deviation; SN, salience network; VAN, ventral attention network.

Additionally, lower DMN‐VAN and greater SN‐CON and VAN‐DAN connectivity were associated with higher initial levels (intercepts) of and steeper rates of change (quadratic slopes) in *p*‐factor scores over wave. These steeper rates of quadratic change were significant over and above the linear rates of change. To illustrate these quadratic interactions, simple slopes were estimated at the mean and +/− 1 standard deviation from the mean using the emmeans package (Lenth, 2025) (Figure 2). Preadolescents with lower baseline DMN‐VAN connectivity had higher initial p‐factor scores at baseline and showed the steepest quadratic trajectory of p‐factor scores (*β* = −0.010, 95% CI [−0.010, −0.004]), followed by youth with average (*β* = −0.009, 95% CI [−0.011, −0.006]) and higher (*β* = −0.007, 95% CI [−0.013, −0.008]) DMN‐VAN connectivity (all *p* < 0.001). Preadolescents with higher baseline SN‐CON and VAN‐DAN connectivity had higher initial p‐factor scores and showed the steepest quadratic trajectory of p‐factor scores (SN‐CON: *β* = −0.009, 95% CI [−0.012, −0.006]; VAN‐DAN: *β* = −0.011, 95% CI [−0.013, −0.008]), followed by youth with average (both networks: *β* = −0.009, 95% CI [−0.011, −0.006]) and lower (SN‐CON: *β* = −0.008, 95% CI [−0.011, −0.005]; VAN‐DAN: *β* = −0.006, 95% CI [−0.009, −0.004]) connectivity (all *p* < 0.001). Sensitivity analyses revealed these associations remained significant after excluding participants with mean FD > 0.2 mm, controlling for medication use, and excluding participants with completely missing CBCL follow‐up data (Supporting Information S1; Table S7).

## DISCUSSION

In the ABCD study, we examined prospective relations between baseline RSFC and general psychopathology trajectories over a 3‐year period during the transition to adolescence. First, we identified a significant nonlinear, quadratic trajectory of p‐factor scores over time. Second, consistent with prior research and our hypotheses, we found that lower within‐DMN and within‐DAN connectivity and greater DMN‐CON, DMN‐DAN, and VAN‐CON connectivity were associated with higher between‐person levels of p‐factor scores, and now show that these relations persisted over time, independent of sex, age, scanner, study site, head motion, and medication use. Third, for the first time, we found that lower DMN‐VAN connectivity and greater SN‐CON and VAN‐DAN connectivity prospectively related to steeper within‐person rates of quadratic change in p‐factor scores over time. None of the non‐cognitive RSFC networks were associated with between‐person or within‐person change in p‐factor scores. These findings suggest that alterations in the communication of nodes within and between networks involved in self‐referential processing, top‐down and bottom‐up attention, salience, and cognitive control prospectively relate to general psychopathology trajectories during emerging adolescence.

The significant prospective associations between general psychopathology and RSFC of networks involved in cognitive and attentional processes, but not networks involved in more basic, unimodal functions continues to implicate dysfunctions in higher‐order cognition as a shared mechanism underlying psychopathology broadly. These findings are in line with extensive prior research demonstrating neural alterations in cognitive and attentional regions (Elliott et al., 2018; Moberget et al., 2019; Romer et al., 2018, 2021; Snyder et al., 2017) and poorer performance on cognitive tests in youth high in general psychopathology (Bloemen et al., 2018; Castellanos‐Ryan et al., 2016; Snyder et al., 2019; Wade et al., 2019; White et al., 2017). Additionally, our findings both replicate and extend previous cross‐sectional associations between RSFC and the p‐factor (Karcher et al., 2021; Lees et al., 2021; Sripada et al., 2021; Xia et al., 2018). Specifically, replicating prior ABCD studies (Karcher et al., 2021; Lees et al., 2021; Sripada et al., 2021), we found associations of lower within‐DMN and within‐DAN connectivity and greater DMN‐CON and DMN‐DAN connectivity with higher between‐person levels of p‐factor scores, and we now extend this work by demonstrating these relations persist during preadolescence into early adolescence. Further, lower DMN‐VAN connectivity and greater VAN‐DAN connectivity previously has been associated concurrently with higher p‐factor scores in the ABCD sample (Karcher et al., 2021; Sripada et al., 2021), but we now show that the connectivity between these networks prospectively related to within‐person rates of change in *p* over time.

Many of our findings revolve around the connectivity of the DMN, a network involved in self‐referential processing, suggesting dysfunctions both in the communication of nodes within the DMN and between nodes within the VAN, DAN, and CON networks are prospectively related to general psychopathology trajectories. These findings are consistent with Menon's (2011) triple network model and extensive research demonstrating alterations in DMN connectivity associated with a wide range of mental disorders (Fox et al., 2005; Menon, 2011; Raichle, 2015). Lower within‐DMN connectivity and greater DMN‐CON and DMN‐DAN connectivity could suggest less differentiation of the DMN from other top‐down cognitive and attentional control networks. One hypothesis is that this reduced differentiation may manifest as difficulties disengaging attention to internal, self‐related thoughts in situations requiring cognitive and attentional control, which our findings suggest could be a marker of persistent general psychopathology. We also newly found that greater VAN‐CON connectivity was associated with higher between‐person levels of p‐factor scores over time. This finding suggests that less differentiation of the VAN and CON networks involved in bottom‐up attention and top‐down cognitive control may be associated with higher persistent general psychopathology.

The hypothesis that a lack of differentiation between these cognitive and attentional networks may be associated with general psychopathology trajectories is consistent with studies on the neurodevelopment of these networks during childhood and adolescence. Specifically, patterns of neural connectivity evolve during development—network segregation (i.e., nodes between networks decrease co‐activation as networks functionally specialize) and network integration (i.e., nodes within networks increase co‐activation) both improve with age in non‐clinical samples (Grayson & Fair, 2017; Gu et al., 2015; Morgan et al., 2018; Sherman et al., 2014; Stevens, 2009). In typically‐developing adolescents, evidence suggests that nodes within the DMN become increasingly connected, whereas the rest of the cognitive control networks (i.e., FPN, SN, VAN, DAN, CON) become increasingly segregated from one another (Gu et al., 2015; Sherman et al., 2014). In line with this, increased cognitive abilities are associated with greater segregation of higher‐order cognitive networks and integrated DMN throughout adolescence (Gu et al., 2015; Satterthwaite et al., 2013). Further, our results are consistent with prior research that found delayed maturation of the DMN was associated with general psychopathology in childhood (Sato et al., 2016). Thus, one hypothesis is that early failure for these functional networks to properly form connections and appropriately integrate and segregate during childhood is what drives increases in psychopathology broadly. However, this hypothesis will need to be directly tested in future studies examining the development of these networks as prospective predictors of changes in general psychopathology.

In terms of the trajectory of general psychopathology, we found that p‐factor scores demonstrated a significant quadratic developmental trajectory from preadolescence to emerging adolescence. Specifically, p‐factor scores decreased during preadolescence (baseline to wave 3; ages 9–12) and began increasing during the transition to adolescence (wave 3 to wave 4; ages 12–13). Decreases in the p‐factor during preadolescence is consistent with prior ABCD research that demonstrated CBCL subscale scores (internalizing, externalizing) decreased from baseline to wave 3 (Barch et al., 2021). Indeed, psychopathology may truly decline during preadolescence before the onset or worsening of many forms of mental disorder in adolescence (Kessler et al., 2005; McGrath et al., 2020; Solmi et al., 2022). For example, anxiety and impulse‐control disorders typically onset in preadolescence and then begin to decrease before substance use, mood, and thought disorders onset in adolescence and young adulthood (American Psychiatric Association, 2013; McLaughlin & King, 2015), consistent with p‐factor scores beginning to increase from waves 3–4 (ages 12–13). Further, increases in the p‐factor during emerging adolescence are consistent with findings from the Generation *R* study, which found that the majority of children switched between psychopathology subgroups (i.e., internalizing, externalizing, and dysregulated) during the transition from childhood to adolescence (Blok et al., 2022). Prior longitudinal research also has demonstrated evidence for *p*‐differentiation (i.e., more distinct forms of psychopathology with lower rates of comorbidity) in early adolescence, followed by evidence of dynamic mutualism (i.e., increases in comorbidity due to causal interactions among psychopathology symptoms) in middle adolescence through young adulthood (McElroy et al., 2018; Richards et al., 2024). That DMN‐VAN, VAN‐DAN, and SN‐CON connectivity prospectively related to the rate of the quadratic p‐factor trajectory could suggest that aberrant connectivity between these cognitive and attentional networks might represent a childhood marker of risk for steeper increases in general psychopathology during the adolescent vulnerability period.

However, the effect sizes of relations between RSFC and p‐factor scores over time were small, particularly the associations between RSFC and within‐person changes in *p*, possibly because the influence of RSFC on future general psychopathology may increase with time as psychopathology continues to emerge in later years. We examined associations over a relatively short 3‐year timeframe during pre‐ and early adolescence, prior to the onset of most mental disorders during adolescence and young adulthood (Kessler et al., 2005; McGrath et al., 2020; Solmi et al., 2022). Consequently, there was not much change or variability in the quadratic slopes observed in the p‐factor scores over the waves, restricting the ability to prospectively predict such change. These associations may strengthen as more forms of disorder onset into mid‐ and later adolescence.

Our study has the following limitations. First, approximately 17% of included data had missing p‐factor scores at one or more follow‐up waves, with significant differences between those with complete versus missing data. However, sensitivity analyses demonstrated results were robust to the removal of participants with completely missing follow‐up data. Second, *p*‐factor scores were calculated based on parent‐report data, which may introduce reporter bias. In future study waves, adolescent self‐report data will be available and should be examined. Third, we examined patterns of connectivity among entire networks (i.e., averaging pairwise correlations among all nodes within and between networks), which does not allow for determining which specific nodes may be driving the increases or decreases in network RSFC related to the p‐factor; this should be tested in future studies. Fourth, although results broadly support the hypothesis that failure for networks to appropriately integrate and segregate in pre‐adolescence may be driving the trajectory of psychopathology, these analyses did not examine RSFC over time and thus cannot determine whether these early alterations in connectivity truly signal delayed or atypical patterns of network integration or segregation. Determining how the development of RSFC networks relates to trajectories of psychopathology is an important future direction.

Despite these limitations, this is the first study to examine whether alterations in RSFC prospectively related to between‐person differences and within‐person changes in p‐factor scores during the transition to adolescence. Our novel findings identified specific aberrant patterns of RSFC within and between cognitive and attentional networks that might contribute to general psychopathology development in young people. Dysfunctions in cognitive and attentional control supported by alterations in the communication of the DMN, VAN, DAN, SN, and CON networks may be markers of risk for greater comorbidity and severity of psychopathology during the adolescent vulnerability period. Future studies should test the hypothesis that less differentiation of these networks in childhood may be associated with greater risk for developing multiple forms of mental disorder in adolescence.

## AUTHOR CONTRIBUTIONS

**Jenna Jones Devine**: Conceptualization; writing—original draft; writing—review and editing; visualization; formal analysis. **Garrett R. Hosterman**: Writing—review and editing; formal analysis; visualization. **Jolee Sloss**: Visualization; writing—review and editing; formal analysis. **Adrienne L. Romer.** Conceptualization; writing—original draft; writing—review and editing; supervision.

## CONFLICT OF INTEREST STATEMENT

The authors declare no conflicts of interest.

## ETHICAL CONSIDERATIONS

Institutional review board approval was obtained by a central Institutional Review Board at the University of California, San Diego for most ABCD research sites, with a few sites obtaining local Institutional Review Board approval (Auchter et al., 2018). Parent(s)/legal guardian(s) provided written informed consent, and children assented before participation in the study in accordance with each data collection site's Institutional Review Board. Data were accessed through the National Institute of Mental Health Data Archive. As the present study used existing anonymized data, no additional ethical approval was required.

## Supporting information

Supporting Information S1

## Data Availability

The data that support the findings of this study are openly available in NIMH Data Archive at http://dx.doi.org/10.15154/z563‐zd24, reference number 2313.
