# Supplementary material for: Alterations in resting‐state functional connectivity relate to psychopathology trajectories during emerging adolescence
Source: JCPP Adv. 2026 May 30:e70135. Online ahead of print. doi: 10.1002/jcv2.70135 (PMC13339399; doi:10.1002/jcv2.70135)

**Alterations in resting-state functional connectivity relate to psychopathology trajectories during emerging adolescence**

**Supporting Information**

**Appendix S1. MRI Acquisition, Processing, and Quality Control**

A detailed description of full imaging acquisition, scanning, processing, and quality assurance procedures has been reported elsewhere (Casey et al., 2018; Hagler et al., 2019). Briefly, imaging protocols, procedures, and processing were developed and harmonized across 21 ABCD study sites and five scanner platforms by the ABCD Data Analysis, Informatics, and Resources Center (DAIRC) and ABCD Imaging Acquisition Workgroup. Resting-state functional MRI data was collected using 3T scanners (Siemens Prisma and Prisma Fit, General Electric MR 750, Philips Achieva dStream and Ingenia) in two sets of two 5-minute runs (i.e., 20 minutes total) while participants looked at a fixation cross on the screen. Specific scan parameters are as follows: TR 800 ms; TE 30 ms; FOV 216 x 216; FOV phase of 100%; matrix 90 x 90; 60 slices; flip angle of 52°; voxel resolution of 2.4×2.4×2.4 mm. For sites that use Siemens scanners, fMRI Integrated Real-time Motion Monitor (FIRMM) was used to detect real-time motion during resting-state scans and allow operators to adjust scanning parameters as needed to help minimize motion.

After data collection, the DAIRC performed centralized pre-processing and minimal analysis of all resting-state data. First, head motion was corrected by registering each frame to the first. Additionally, B_0_ and gradient nonlinearities distortion corrections were performed. After fMRI preprocessing, data were further processed pre-analysis, including removal of initial frames and time series correlated with motion, and normalization to remove quadratic trends. Next, ROI-average time courses were calculated using FreeSurfer’s automated brain segmentation and resampled to align with fMRI data. These preprocessed time courses are then samples onto the cortical surface for each subject and the Gordon functional parcellation. Within- and between-network connectivity was calculated by taking correlation values for each pair of ROIs and averaged the Fisher transformed z-statistics and averaged across the 13 networks defined in the Gordon parcellation. Participants were excluded from the present study if they failed at least one fMRI quality assurance check, including: failing T1w QC or not having T1w registration available, B0 unwarp unavailable, less than 375 rsfMRI frames after censoring, failing the ventral and dorsal field of view cutoff, or failing FreeSurfer QC.

**Appendix S2. Confirmatory Factor Analyses**

Confirmatory factor analyses (CFAs) are detailed elsewhere (Romer et al., 2023; Romer & Pizzagalli, 2021). Briefly, higher-order models of the structure of psychopathology were fit at each of the four waves using CBCL items. We used the higher-order factor structure from Michelini et al. (2019), which identified five lower-order factors (externalizing, internalizing, neurodevelopmental, somatization, and detachment) and a higher-order p factor.

(Michelini et al., 2019) found that the following 31 CBCL items either did not load on any factor or were low frequency items (<0.5% rated as 1 or 2), which we excluded from our CFAs: “Drinks alcohol without parents' approval”, “Sexual problems”, “Smokes, chews, or sniffs tobacco”, “Truancy, skips school”, “Uses drugs for non-medical purposes (don't include alcohol or tobacco)”, “Plays with own sex parts in public,” “Play with own sex parts too much,” “Overeating,” “Overweight,” “Hears sounds or voices that aren’t there,” “Sees things that aren’t there,” “Bowel movements outside toilet,” “Trouble sleeping,” “Wets self during the day,” “Wets the bed,” “Wishes to be of opposite sex,” “Clings to adult or too dependent,” “Cries a lot,” “Doesn’t eat well,” “Gets teased a lot,” “Bites fingernails,” “Nightmares,” “Constipated, doesn’t move bowels,” “Picks nose, skin, or other parts of body,” “Prefers being with older kids,” “Sleeps less than most kids,” “Sleeps more than most kids,” “Speech problem,” “Stores up too many things he/she doesn’t need,” “Talks or walks in sleep,” and “Thumb-sucking”. They also found that the following 10 CBCL items cross-loaded on more than one factor, which we also excluded to create a more stringent criterion for identifying p: “Secretive, keeps things to self”, “Strange behavior,” “There is very little he/she enjoys,” “Unhappy, sad, or depressed,” “Unusually loud,” “Deliberatively harms self or attempts suicide,” “Feels or complains that no one loves him/her,” “Impulsive or acts without thinking,” “Talks about killing self,” and “Overtired without good reason”.

Michelini et al. (2019) created the following six composite scores of items that were highly correlated (*r*>0.75), which we retained in our analyses: Composite 1 (“Physically attacks people” + “Threatens people”); Composite 2 (“Disobedient at home” + “Disobedient at school” + “Breaks rules at home, school, or elsewhere”); Composite 3 (“Destroys his/her own things” + “Destroys things belonging to his/her family or others” + “Vandalism”); Composite 4 (“Steals at home” + “Steals outside the home”); Composite 5 (“Doesn’t get along with other kids” + “Not liked by other kids”); Composite 6 (“Can’t concentrate, can’t pay attention for long” + “Inattentive or easily distracted” + “Can’t sit still, restless, or hyperactive”).

We removed 3 additional CBCL items for cross-loading on more than one factor (“Showing off or clowning,” “Strange ideas,” and “Talks too much”), 4 items that did not have enough variability (“Thinks about sex too much,” “Sets fires,” “Self-conscious or easily embarrassed,” and “Cruel to animals”), and one item that resulted in a non-positive definite solution (“Other physical problems without known physical cause”) at one or more of the waves. This left a total of 60 CBCL items/composites entered into the CFAs at each wave.

The CFAs were performed in Mplus version 8.6 (Muthen & Muthen, 1998) using the weighted least squares means and variance adjusted (WLSMV) algorithm (see Table S1). Analysis code is available at https://github.com/Ageyr13/ABCD_RSFC_MLM.git. The WLSMV estimator is appropriate for categorical and nonmultivariate normal data and provides consistent estimates when data are missing at random with respect to covariates (Asparouhov & Muthén, 2010). We computed standard errors using “TYPE=COMPLEX” in Mplus, which uses a sandwich estimator to account for non-independence of observations due to cluster sampling within family. Model fit was assessed using the chi-square value, comparative fit index (CFI), Tucker-Lewis index (TLI), and root-mean square error of approximation (RMSEA). Nonsignificant chi-square tests indicate good model fit; nonetheless, this test generally is overpowered in large sample sizes. CFI and TLI>0.90 indicate adequate fit; RMSEA<0.08 is considered acceptable (Kline, 2015).

**Appendix S3. Longitudinal Measurement Invariance**

We previously demonstrated metric and scalar invariance of the higher-order factor model over the first three ABCD study waves (Romer et al., 2023) indicating that the factors are equivalently measured over time. Here, in the present study, we additionally tested the longitudinal measurement over all four study waves. We tested models of configural, metric, and scalar invariance. Configural invariance tests whether the same factor structure can adequately fit the data over time. To do this, we tested a model in which the CBCL items loaded on the same factor at each wave. Metric invariance tests whether the factor loadings are equivalent over time. If metric invariance holds, we can examine change in relative status of participants over time using residualized change models. We tested a model in which the factor loadings were equivalent across the three waves. Scalar invariance tests whether the factor loadings and intercepts are equivalent over time. If scalar invariance holds, we can examine within-person change. As CBCL items were treated as ordinal and WLSMV estimation was employed, scalar invariance tests equivalence of response category thresholds rather than intercepts. Therefore, for this test, we equated both the factor loadings and thresholds of each CBCL item over the four waves. We used likelihood ratio testing to compare the fit of these three models (using “DIFFTEST” function in Mplus for WLSMV estimation), which tested whether adding additional equality constraints resulted in a significant decrement to model fit.

Conducting a direct test of longitudinal measurement invariance of the higher-order model did not converge using WLSMV estimation. Therefore, we tested the invariance of the lower-order psychopathology factors in separate models (Table S2). Chi-square difference tests have the power to detect inconsequential differences between groups in highly complex models such as ours; thus, invariance was defined as a ΔCFI<0.01 and ΔRMSEA<0.007 between the scalar and configural models (Neufeld et al., 2024). Likelihood ratio testing showed that imposing metric invariance did not result in a significant decrement in model fit relative to configural invariance. Similarly, imposing scalar invariance did not result in significant decrement in model fit relative to configural invariance. In terms of measurement invariance of the p-factor, Table S3 shows that the p-factor loadings are highly similar across waves. To provide a test of metric invariance of the p-factor, we fixed p-factor loadings in the wave 2, 3, and 4 factor models to be equivalent to the loadings from the baseline model and we compared model fit. Although not a formal test of metric invariance, fit statistics were highly similar between these models, suggesting that equating the baseline p-factor loadings to be the same in the follow-up waves did not worsen model fit. These findings support the use of longitudinal multilevel modeling to examine within-person change in p-factor scores over time.

**References**

Asparouhov, T., & Muthén, B. (2010). *Weighted Least Squares Estimation with Missing Data*.

Kline, R. B. (2015). *Principles and Practice of Structural Equation Modeling, Fourth Edition*. Guilford Publications.

Michelini, G., Barch, D. M., Tian, Y., Watson, D., Klein, D. N., & Kotov, R. (2019). Delineating and validating higher-order dimensions of psychopathology in the Adolescent Brain Cognitive Development (ABCD) study. *Translational Psychiatry*, *9*(1), 1–15. https://doi.org/10.1038/s41398-019-0593-4

Muthen, L. K., & Muthen, B. O. (1998). *Mplus User’s Guide. (Eight ed.).* Muthen & Muthen.

Neufeld, S. A. S., St Clair, M., Brodbeck, J., Wilkinson, P. O., Goodyer, I. M., & Jones, P. B. (2024). Measurement Invariance in Longitudinal Bifactor Models: Review and Application Based on the p Factor. *Assessment*, *31*(4), 774–793. https://doi.org/10.1177/10731911231182687

Romer, A. L., & Pizzagalli, D. A. (2021). Is executive dysfunction a risk marker or consequence of psychopathology? A test of executive function as a prospective predictor and outcome of general psychopathology in the adolescent brain cognitive development study®. *Developmental Cognitive Neuroscience*, *51*, 100994. https://doi.org/10.1016/j.dcn.2021.100994

Romer, A. L., Ren, B., & Pizzagalli, D. A. (2023). Brain Structure Relations With Psychopathology Trajectories in the Adolescent Brain Cognitive Development Study. *Journal of the American Academy of Child & Adolescent Psychiatry*. https://doi.org/10.1016/j.jaac.2023.02.002

**Table S1.** *Model Fit Statistics and Unstandardized Factor Loadings for the Higher-Order Factor Model in the ABCD Baseline Sample.*

| **Standardized Factor Loadings and Model Fit** | **p** | **EXT** | **INT** | **ND** | **SOMAT** | **DETACH** |
| --- | --- | --- | --- | --- | --- | --- |
| Loadings |  |  |  |  |  |  |
| Composite 1 (physically attacks & threatens people) |  | 0.441 |  |  |  |  |
| Cruelty, bullying, or meanness to others |  | 0.411 |  |  |  |  |
| Composite 2 (disobedient & breaks rules) |  | 0.456 |  |  |  |  |
| Gets in many fights |  | 0.412 |  |  |  |  |
| Temper tantrums or hot temper |  | 0.444 |  |  |  |  |
| Argues a lot |  | 0.438 |  |  |  |  |
| Composite 3 (destroys things & vandalism) |  | 0.438 |  |  |  |  |
| Screams a lot |  | 0.422 |  |  |  |  |
| Doesn’t seem to feel guilty after misbehaving |  | 0.401 |  |  |  |  |
| Swearing or obscene language |  | 0.361 |  |  |  |  |
| Teases a lot |  | 0.379 |  |  |  |  |
| Composite 4 (steals at home & outside home) |  | 0.391 |  |  |  |  |
| Stubborn, sullen, or irritable |  | 0.440 |  |  |  |  |
| Lying or cheating |  | 0.396 |  |  |  |  |
| Runs away from home |  | 0.410 |  |  |  |  |
| Sudden changes in mood or feelings |  | 0.453 |  |  |  |  |
| Easily jealous |  | 0.395 |  |  |  |  |
| Composite 5 (doesn’t get along & not liked by other kids) |  | 0.429 |  |  |  |  |
| Suspicious |  | 0.420 |  |  |  |  |
| Demands a lot of attention |  | 0.419 |  |  |  |  |
| Hangs around with others who get in trouble |  | 0.314 |  |  |  |  |
| Feels others are out to get him/her |  | 0.410 |  |  |  |  |
| Sulks a lot |  | 0.417 |  |  |  |  |
| Bragging, boasting |  | 0.304 |  |  |  |  |
| Whining |  | 0.361 |  |  |  |  |
| Too fearful or anxious |  |  | 0.469 |  |  |  |
| Worries a lot |  |  | 0.441 |  |  |  |
| Feels he/she has to be perfect |  |  | 0.299 |  |  |  |
| Feels too guilty |  |  | 0.425 |  |  |  |
| Nervous, high-strung, or tense |  |  | 0.492 |  |  |  |
| Fears he/she might think or do something bad |  |  | 0.403 |  |  |  |
| Feels worthless or inferior |  |  | 0.478 |  |  |  |
| Fears going to school |  |  | 0.410 |  |  |  |
| Fears certain animals, situations, or places, other than school |  |  | 0.335 |  |  |  |
| Complains of loneliness |  |  | 0.455 |  |  |  |
| Composite 6 (can’t concentrate, inattentive, easily distracted, & can’t sit still, restless, or hyperactive) |  |  |  | 0.351 |  |  |
| Daydreams or gets lost in his/her thoughts |  |  |  | 0.266 |  |  |
| Confused or seems to be in a fog |  |  |  | 0.310 |  |  |
| Poorly coordinated or clumsy |  |  |  | 0.292 |  |  |
| Nervous movements or twitching |  |  |  | 0.280 |  |  |
| Fails to finish things he/she starts |  |  |  | 0.336 |  |  |
| Can’t get his/her mind off certain thoughts (obsessions) |  |  |  | 0.335 |  |  |
| Poor schoolwork |  |  |  | 0.308 |  |  |
| **Standardized Factor Loadings and Model Fit** | **p** | **EXT** | **INT** | **ND** | **SOMAT** | **DETACH** |
| Repeats certain acts over and over (compulsions) |  |  |  | 0.325 |  |  |
| Acts too young for his/her age |  |  |  | 0.280 |  |  |
| Gets hurt a lot, accident prone |  |  |  | 0.239 |  |  |
| Prefers being with younger kids |  |  |  | 0.246 |  |  |
| Nausea, feels sick |  |  |  |  | 0.694 |  |
| Stomachaches |  |  |  |  | 0.623 |  |
| Vomiting, throwing up |  |  |  |  | 0.501 |  |
| Headaches |  |  |  |  | 0.527 |  |
| Aches or pains (not stomach/head) |  |  |  |  | 0.532 |  |
| Feels dizzy or lightheaded |  |  |  |  | 0.641 |  |
| Problems with eyes |  |  |  |  | 0.439 |  |
| Rashes or other skin problems |  |  |  |  | 0.403 |  |
| Withdrawn, doesn’t get involved with others |  |  |  |  |  | 0.566 |
| Would rather be alone |  |  |  |  |  | 0.446 |
| Too shy or timid |  |  |  |  |  | 0.366 |
| Refuses to talk |  |  |  |  |  | 0.509 |
| Underactive, slow moving, or lacks energy |  |  |  |  |  | 0.491 |
| EXT | 1.465 |  |  |  |  |  |
| INT | 1.357 |  |  |  |  |  |
| ND | 2.069 |  |  |  |  |  |
| SOMAT | 0.691 |  |  |  |  |  |
| DETACH | 1.169 |  |  |  |  |  |
| Model Fit |  |  |  |  |  |  |
| *X*^2^/df | 22684.017/1705 | | | | | |
| CFI | 0.925 | | | | | |
| TLI | 0.922 | | | | | |
| RMSEA [90% CI] | 0.032 [0.032, 0.033] | | | | | |

*Note.* Unstandardized factor loadings and model fit statistics for the higher-order confirmatory factor analysis at the baseline wave are shown (N=11870; 9850 clusters). All loadings were positive and statistically significant (*p*<0.001). CFI=Comparative Fit Index; CI=confidence interval; DETACH=Detachment; df=degrees of freedom; EXT=Externalizing; INT=Internalizing; ND=Neurodevelopmental; RMSEA=Root Mean Square Error of Approximation; SOMAT=Somatization; TLI=Tucker Lewis Index; *X*^2^=chi-square value.

**Table S2.** *Test of Longitudinal Measurement Invariance of the Lower-Order Psychopathology Factors from the Higher-Order Model.*

| **Model** | **Test of Overall Fit** | | | **Fit Indices** | | | | **LRT Relative to Prior Model** | |
| --- | --- | --- | --- | --- | --- | --- | --- | --- | --- |
|  | *X*^2^ | df | P-value | RMSEA | CFI | TLI | SRMR | ΔRMSEA | ΔCFI |
| **EXT** |  |  |  |  |  |  |  |  |  |
| Configural | 26632.323 | 4699 | <0.001 | 0.020 | 0.957 | 0.955 | 0.048 |  |  |
| Metric | 20953.704 | 4771 | <0.001 | 0.017 | 0.968 | 0.967 | 0.050 | 0.003 | 0.011 |
| Scalar | 21866.828 | 4842 | <0.001 | 0.017 | 0.967 | 0.966 | 0.050 | 0.000 | 0.001 |
| **INT** |  |  |  |  |  |  |  |  |  |
| Configural | 6388.742 | 674 | <0.001 | 0.027 | 0.970 | 0.965 | 0.044 |  |  |
| Metric | 4939.150 | 701 | <0.001 | 0.023 | 0.978 | 0.975 | 0.044 | 0.004 | 0.008 |
| Scalar | 5237.932 | 728 | <0.001 | 0.023 | 0.976 | 0.974 | 0.044 | 0.000 | 0.002 |
| **ND** |  |  |  |  |  |  |  |  |  |
| Configural | 14244.704 | 1002 | <0.001 | 0.033 | 0.956 | 0.951 | 0.055 |  |  |
| Metric | 11310.895 | 1035 | <0.001 | 0.029 | 0.966 | 0.963 | 0.056 | 0.004 | 0.010 |
| Scalar | 11838.807 | 1068 | <0.001 | 0.029 | 0.964 | 0.962 | 0.056 | 0.000 | 0.002 |
| **SOMAT** |  |  |  |  |  |  |  |  |  |
| Configural | 1949.357 | 410 | <0.001 | 0.018 | 0.984 | 0.980 | 0.042 |  |  |
| Metric | 1683.767 | 431 | <0.001 | 0.016 | 0.987 | 0.985 | 0.043 | 0.002 | 0.003 |
| Scalar | 1891.057 | 452 | <0.001 | 0.016 | 0.985 | 0.983 | 0.043 | 0.000 | 0.002 |
| **DETACH** |  |  |  |  |  |  |  |  |  |
| Configural | 1063.773 | 134 | <0.001 | 0.024 | 0.988 | 0.982 | 0.033 |  |  |
| Metric | 924.337 | 146 | <0.001 | 0.021 | 0.990 | 0.986 | 0.034 | 0.003 | 0.002 |
| Scalar | 1339.503 | 158 | <0.001 | 0.025 | 0.984 | 0.981 | 0.035 | 0.004 | 0.006 |

*Note.* Likelihood ratio testing (LRT) was used to compare the fit of models testing configural, metric, and scalar longitudinal invariance across the four waves. Tests of overall model fit, LRT relative to the prior model, and fit indices are shown for each model. Invariance was defined as a ΔCFI<0.01 and ΔRMSEA<0.007 between the scalar and configural models (Neufeld et al., 2024). *X*^2^=chi-square; df=degrees of freedom; RMSEA=Root Mean Square Error of Approximation; CFI=Comparative Fit Index; TLI=Tucker Lewis Index; SRMR=Standardized Root Mean Residual; EXT=Externalizing; INT=Internalizing; ND=Neurodevelopmental; SOMAT=Somatization; DETACH=Detachment.

**Table S3.** *Comparison of Goodness of Fit Indices between Higher-Order Factor Models with P-Factor Loadings Unconstrained versus Constrained Across Waves.*

|  | **Wave 2** | | **Wave 3** | | **Wave 4** | |
| --- | --- | --- | --- | --- | --- | --- |
| **Model Fit** | **Unconstrained** | **Constrained** | **Unconstrained** | **Constrained** | **Unconstrained** | **Constrained** |
| *X*^2^ | 22096.379 | 19581.942 | 21705.944 | 18532.133 | 20898.851 | 18238.284 |
| df | 1705 | 1710 | 1705 | 1710 | 1705 | 1710 |
| CFI | 0.921 | 0.930 | 0.913 | 0.927 | 0.917 | 0.928 |
| TLI | 0.918 | 0.928 | 0.909 | 0.924 | 0.914 | 0.926 |
| RMSEA | 0.033 | 0.031 | 0.033 | 0.030 | 0.033 | 0.031 |
| SRMR | 0.063 | 0.063 | 0.065 | 0.066 | 0.066 | 0.067 |

*Note.* P-factor loadings in the wave 2, 3, and 4 models were constrained to be the same as the p-factor loadings in the baseline wave 1 higher-order model. Although not a formal test of metric invariance (equating factor loadings over time), comparison of fit statistics between the unconstrained and constrained models shows that constraining the p-factor loadings did not worsen model fit. *X*^2^=chi-square value; df=degrees of freedom; CFI=Comparative Fit Index; TLI=Tucker Lewis Index; RMSEA=Root Mean Square Error of Approximation; SRMR=Standardized Root Mean Residual.

**Table S4.** *Bivariate Correlations between All Baseline Wave 1 Study Variables.*

|  | Sex | Age | Achieva | Discovery | Ingenia | Prisma | Mean FD | Medication | P-Factor Scores |
| --- | --- | --- | --- | --- | --- | --- | --- | --- | --- |
| Sex | 1 |  |  |  |  |  |  |  |  |
| Age | **-0.033** | 1 |  |  |  |  |  |  |  |
| Achieva | -0.002 | **-0.027** | 1 |  |  |  |  |  |  |
| Discovery | 0.008 | **-0.061** | **-0.146** | 1 |  |  |  |  |  |
| Ingenia | 0.014 | 0.046 | **-0.052** | **-0.111** | 1 |  |  |  |  |
| Prisma | -0.019 | 0.016 | **-0.167** | **-0.355** | **-0.128** | 1 |  |  |  |
| Mean FD | **-0.068** | **-0.113** | 0.002 | -0.018 | **0.032** | **-0.067** | 1 |  |  |
| Medication | **-0.039** | 0.001 | **-0.41** | 0.019 | -0.007 | **0.023** | -0.020 | 1 |  |
| P-factor scores | **-0.101** | -0.010 | **-0.033** | 0.012 | **-0.032** | **0.036** | **0.057** | **0.146** | 1 |
| DMN-DMN | **0.147** | **0.075** | 0.004 | **-0.216** | **-0.081** | **0.116** | **-0.214** | -0.004 | **-0.050** |
| DMN-FPN | **-0.035** | -0.011 | -0.007 | **-0.022** | **0.050** | **-0.032** | **0.079** | 0.019 | **0.031** |
| DMN-SN | **0.027** | -0.002 | **-0.036** | **-0.068** | 0.020 | **0.048** | **0.022** | 0.007 | 0.009 |
| DMN-VAN | **0.080** | 0.005 | **-0.086** | **-0.097** | **-0.057** | **0.051** | **-0.075** | 0.006 | **-0.041** |
| DMN-DAN | **-0.105** | **-0.082** | **0.050** | **0.145** | **0.126** | **-0.155** | **0.242** | 0.013 | **0.072** |
| DMN-CON | **-0.156** | **-0.084** | **-0.046** | **0.030** | **0.083** | **-0.066** | **0.241** | **0.024** | **0.062** |
| DMN-AD | **-0.108** | -0.018 | **-0.120** | **-0.094** | **-0.037** | **0.047** | **0.051** | **0.043** | 0.004 |
| DMN-CA | **0.098** | -0.019 | **0.042** | **-0.032** | **0.052** | -0.008 | 0.000 | 0.010 | 0.008 |
| DMN-N | **0.049** | **0.024** | **-0.062** | **-0.313** | **-0.152** | **0.195** | **-0.189** | 0.021 | -0.008 |
| DMN-RSPLTP | **0.047** | **0.024** | 0.005 | **-0.048** | **-0.057** | -0.008 | -0.012 | 0.007 | -0.005 |
| DMN-SMH | **-0.101** | **-0.030** | **-0.123** | -0.013 | **-0.066** | **0.033** | **0.035** | **0.040** | **0.035** |
| DMN-SMM | **-0.102** | -0.003 | **-0.115** | -0.005 | -0.007 | **-0.023** | **0.056** | **0.026** | 0.003 |
| DMN-VS | **-0.102** | **-0.057** | **0.040** | **0.203** | **0.066** | **-0.150** | **0.172** | 0.003 | 0.013 |
| FPN-FPN | **0.102** | **0.053** | **0.061** | **-0.148** | 0.003 | **0.023** | **-0.127** | **-0.028** | **-0.027** |
| FPN-SN | **-0.099** | **0.049** | -0.004 | **-0.198** | 0.005 | **0.068** | **-0.027** | **-0.038** | 0.011 |
| FPN-VAN | -0.004 | 0.007 | **0.099** | **-0.113** | 0.011 | -0.004 | **0.038** | **0.030** | **0.023** |
| FPN-DAN | **0.078** | -0.002 | **0.129** | **0.025** | **0.141** | **-0.112** | 0.003 | -0.014 | **-0.030** |
| FPN-CON | **-0.045** | **-0.035** | **0.023** | **-0.083** | **0.081** | -0.015 | **0.117** | -0.002 | **0.022** |
| FPN-AD | **-0.075** | 0.000 | **0.028** | **-0.117** | -0.002 | **0.027** | **0.037** | **0.057** | 0.002 |
| FPN-CA | 0.003 | 0.009 | **-0.039** | 0.013 | -0.006 | -0.019 | -0.010 | **-0.037** | 0.004 |
| FPN-N | **0.081** | -0.018 | **-0.066** | **-0.060** | **-0.166** | **0.029** | **-0.079** | 0.008 | 0.002 |
| FPN-RSPLTP | -0.003 | -0.014 | **-0.056** | **0.080** | 0.016 | **-0.063** | **0.093** | -0.003 | 0.001 |
| FPN-SMH | **-0.030** | **-0.052** | -0.008 | 0.010 | **0.028** | **-0.028** | **0.070** | **0.063** | 0.004 |
| FPN-SMM | **-0.054** | **-0.024** | **0.032** | **-0.044** | **0.070** | **-0.043** | **0.092** | **0.032** | 0.008 |
| FPN-VS | **-0.036** | **-0.041** | **0.079** | **0.183** | **0.081** | **-0.101** | **0.091** | -0.007 | 0.004 |
| SN-SN | -0.004 | 0.003 | -0.005 | **-0.156** | 0.018 | **0.091** | **-0.077** | **-0.032** | 0.013 |
| SN-VAN | -0.012 | -0.019 | **0.030** | **-0.163** | **0.031** | **0.057** | **0.063** | **0.025** | 0.004 |
| SN-DAN | **-0.080** | 0.010 | **0.035** | **-0.022** | **0.028** | **-0.081** | **0.092** | 0.011 | 0.001 |
| SN-CON | **-0.024** | -0.019 | 0.019 | **-0.104** | **0.048** | 0.004 | **0.034** | 0.012 | **0.027** |
| SN-AD | **-0.056** | **-0.035** | **0.027** | **-0.097** | 0.001 | 0.003 | **0.119** | **0.058** | **0.025** |
| SN-CA | **-0.040** | 0.020 | **-0.073** | **-0.055** | **-0.054** | **0.071** | **-0.063** | **-0.043** | 0.008 |
| SN-N | **0.066** | **-0.067** | -0.017 | **-0.162** | **0.102** | **0.028** | **0.050** | **0.023** | -0.011 |
| SN-RSPLTP | **-0.045** | **-0.049** | **-0.043** | **0.029** | 0.001 | **-0.052** | **0.200** | 0.003 | 0.011 |
| SN-SMH | 0.015 | **-0.026** | **-0.056** | -0.004 | **-0.037** | -0.017 | **0.092** | **0.078** | **0.025** |
| SN-SMM | **-0.029** | **-0.035** | 0.007 | **-0.026** | 0.009 | **-0.031** | **0.084** | **0.048** | 0.014 |
| SN-VS | **-0.065** | **-0.049** | -0.011 | **0.127** | **-0.068** | **-0.070** | **0.073** | 0.006 | -0.007 |
| VAN-VAN | -0.010 | 0.010 | -0.013 | **-0.153** | **-0.063** | **0.076** | **-0.087** | 0.019 | -0.006 |
| VAN-DAN | **-0.031** | -0.014 | **0.131** | 0.009 | **0.119** | **-0.088** | **0.132** | 0.004 | **0.047** |
| VAN-CON | **-0.116** | -0.009 | **0.075** | **-0.190** | **0.087** | **0.030** | **0.105** | **0.039** | **0.046** |
| VAN-AD | **-0.078** | **-0.033** | 0.021 | **-0.194** | **0.047** | **0.054** | **0.067** | **0.028** | **0.031** |
| VAN-CA | -0.001 | **-0.042** | -0.011 | -0.018 | **0.031** | 0.011 | **0.053** | 0.012 | -0.016 |
| VAN-N | **0.037** | **-0.022** | **-0.090** | **-0.204** | **-0.052** | **0.109** | **-0.056** | 0.015 | -0.016 |
| VAN-RSPLTP | **-0.068** | **-0.052** | **-0.044** | **0.049** | 0.007 | **-0.071** | **0.214** | -0.006 | 0.016 |
| VAN-SMH | **-0.059** | 0.001 | **-0.039** | **-0.074** | -0.005 | **0.055** | **-0.062** | 0.002 | **0.026** |
| VAN-SMM | **-0.063** | 0.007 | 0.020 | **-0.141** | **0.068** | -0.004 | **0.040** | 0.001 | 0.011 |
| VAN-VS | **-0.070** | **-0.029** | **0.100** | **0.134** | **0.085** | **-0.139** | **0.125** | **-0.030** | 0.005 |
| DAN-DAN | **0.026** | **0.066** | 0.008 | **-0.089** | -0.017 | **0.067** | **-0.156** | -0.009 | **-0.045** |
| DAN-CON | **0.035** | **0.067** | 0.006 | **-0.102** | 0.001 | 0.018 | **-0.027** | -0.003 | **-0.024** |
| DAN-AD | **0.040** | **0.025** | **0.046** | **-0.080** | **0.066** | 0.000 | **0.076** | -0.004 | 0.015 |
| DAN-CA | **-0.058** | 0.019 | **-0.040** | -0.013 | 0.017 | -0.010 | 0.017 | 0.004 | -0.020 |
| DAN-N | -0.017 | **-0.034** | -0.011 | **0.182** | **0.035** | **-0.134** | **0.142** | -0.011 | **0.025** |
| DAN-RSPLTP | **-0.036** | 0.018 | **-0.105** | **-0.071** | -0.010 | **0.111** | **-0.050** | 0.004 | 0.000 |
| DAN-SMH | **0.053** | 0.007 | **0.047** | **-0.032** | **0.078** | **-0.023** | **0.059** | 0.003 | -0.017 |
| DAN-SMM | **0.023** | -0.006 | **0.114** | **-0.070** | **0.155** | -0.003 | **0.094** | -0.014 | **0.022** |
| DAN-VS | 0.015 | **0.039** | -0.021 | **-0.083** | -0.004 | **0.142** | **-0.098** | 0.007 | -0.012 |
| CON-CON | **0.107** | **0.054** | **-0.033** | **-0.206** | **-0.025** | **0.128** | **-0.197** | **0.031** | **-0.027** |
| CON-AD | **0.063** | 0.010 | 0.012 | **-0.175** | **0.055** | **0.051** | -0.011 | **0.046** | 0.007 |
| CON-CA | **-0.071** | **0.028** | **-0.105** | **-0.031** | **-0.086** | **0.059** | **-0.032** | -0.020 | **-0.023** |
| CON-N | **-0.091** | **-0.053** | **-0.028** | 0.013 | **0.134** | **-0.085** | **0.192** | 0.008 | **0.024** |
| CON-RSPLTP | **-0.140** | **-0.053** | **-0.066** | -0.007 | 0.013 | **-0.024** | **0.200** | **-0.022** | **0.028** |
| CON-SMH | **0.088** | **0.048** | **-0.048** | **-0.115** | **-0.031** | **0.065** | -0.004 | **0.029** | 0.003 |
| CON-SMM | **0.043** | 0.009 | **0.034** | **-0.146** | **0.053** | **0.065** | **-0.052** | 0.012 | 0.014 |
| CON-VS | 0.002 | -0.001 | **0.034** | **0.044** | -0.021 | -0.010 | **-0.038** | **-0.031** | **-0.027** |
| AD-AD | **0.050** | 0.000 | 0.002 | **-0.181** | **0.094** | **0.046** | **-0.073** | -0.011 | **-0.024** |
| AD-CA | **-0.107** | -0.003 | **-0.080** | **-0.053** | **-0.077** | **0.050** | **0.069** | **0.029** | 0.017 |
| AD-N | **-0.089** | -0.006 | **-0.046** | **-0.150** | **0.062** | 0.016 | **0.053** | 0.018 | -0.011 |
| AD-RSPLTP | **-0.143** | **-0.045** | **-0.043** | **-0.033** | -0.013 | -0.011 | **0.197** | **-0.023** | **0.025** |
| AD-SMH | **0.076** | **0.038** | **-0.039** | **-0.089** | 0.020 | **0.053** | **-0.115** | **-0.054** | -0.008 |
| AD-SMM | **0.032** | **0.024** | **-0.034** | **-0.131** | **0.045** | **0.048** | **-0.109** | **-0.036** | -0.012 |
| AD-VS | **-0.027** | **-0.026** | **0.049** | **0.057** | **0.047** | **-0.054** | **0.101** | **-0.057** | **0.022** |
| CA-CA | -0.016 | **0.071** | **-0.023** | **-0.023** | **-0.060** | **0.048** | **-0.133** | -0.014 | -0.020 |
| CA-N | **0.085** | **-0.040** | 0.021 | **-0.022** | **-0.027** | -0.005 | 0.006 | -0.005 | -0.011 |
| CA-RSPLTP | **0.077** | 0.007 | **-0.031** | **-0.029** | -0.010 | **0.027** | -0.021 | 0.000 | -0.006 |
| CA-SMH | **-0.020** | -0.010 | **-0.080** | -0.010 | -0.001 | 0.003 | **0.110** | **0.048** | **0.022** |
| CA-SMM | **-0.074** | **-0.031** | **-0.079** | **0.035** | **-0.043** | 0.000 | **0.068** | **0.039** | 0.010 |
| CA-VS | **-0.055** | -0.021 | **-0.031** | -0.014 | -0.019 | **0.034** | **0.049** | 0.014 | 0.017 |
| N-N | **-0.030** | -0.006 | **-0.112** | **-0.347** | **-0.021** | **0.187** | **-0.049** | 0.017 | 0.005 |
| N-RSPLTP | **-0.092** | -0.016 | **0.057** | **-0.072** | 0.010 | **-0.022** | **0.035** | 0.003 | 0.000 |
| N-SMH | **-0.081** | 0.011 | **-0.080** | 0.011 | **-0.057** | **0.021** | **-0.057** | **0.021** | 0.010 |
| N-SMM | **-0.108** | **0.033** | -0.015 | -0.016 | **0.063** | **-0.052** | **0.042** | -0.008 | 0.004 |
| N-VS | **-0.071** | **-0.028** | **0.116** | **0.258** | **0.075** | **-0.177** | **0.162** | **-0.023** | -0.001 |
| RSPLTP-RSPLTP | **0.085** | 0.009 | **-0.071** | **-0.069** | **-0.089** | **0.101** | **-0.232** | 0.012 | **-0.028** |
| RSPLTP-SMH | **-0.143** | -0.003 | **-0.044** | -0.018 | 0.016 | 0.008 | **0.054** | -0.016 | 0.018 |
| RSPLTP-SMM | **-0.087** | **-0.054** | **-0.050** | **0.035** | -0.007 | -0.009 | **0.060** | -0.003 | 0.018 |
| RSPLTP-VS | **0.050** | 0.003 | **-0.101** | **-0.119** | **-0.022** | **0.158** | **-0.069** | **0.022** | 0.003 |
| SMH-SMH | -0.003 | **0.041** | **0.040** | **-0.044** | -0.013 | **0.021** | **-0.099** | **-0.063** | -0.002 |
| SMH-SMM | 0.006 | **0.047** | **0.056** | **-0.056** | **0.062** | 0.013 | **-0.065** | **-0.067** | 0.018 |
| SMH-VS | 0.003 | 0.011 | **0.087** | **0.032** | **0.112** | **-0.058** | **0.037** | **-0.052** | -0.002 |
| SMM-SMM | **-0.029** | **0.025** | **-0.080** | **-0.026** | **-0.144** | **0.093** | **-0.151** | -0.015 | -0.018 |
| SMM-VS | **0.057** | **-0.034** | **0.059** | 0.021 | **0.041** | -0.015 | **0.023** | -0.010 | -0.011 |
| VS-VS | **0.085** | **0.077** | **-0.068** | **-0.299** | -0.021 | **0.211** | **-0.151** | **0.024** | -0.012 |

*Note.* Bivariate correlations between all baseline study variables are shown. Correlations that survived FDR correction for the 900 tests (q<0.05) are bolded. DMN = default mode network; FPN = frontoparietal network; SN = salience network; VAN = ventral attention network; DAN = dorsal attention network; CON = cingulo-opercular network; VS = visual network; AD = auditory network; CA = cingulo-parietal network; RSPLTP = retrospenial temporal network; SMM = sensorimotor mouth network; SMH = sensorimotor hand network; N = “none” network; FD = framewise displacement.

**Table S5.** *Follow-Up Tests of Relations between Resting-State Functional Connectivity and the P-Factor at Follow-up Waves.*

| **Resting-State Functional Connectivity** | **Std. β** | **95% CI** |
| --- | --- | --- |
| **DMN-DMN** |  |  |
| Wave 2 | -0.030* | [-0.054, -0.006] |
| Wave 3 | -0.033* | [-0.060, -0.006] |
| Wave 4 | -0.036* | [-0.066, -0.005] |
| **DMN-VAN** |  |  |
| Wave 2 | -0.037*** | [-0.061, -0.014] |
| Wave 3 | -0.046*** | [-0.072, -0.020] |
| Wave 4 | -0.054*** | [-0.084, -0.024] |
| **DMN-DAN** |  |  |
| Wave 2 | 0.057*** | [0.033, 0.081] |
| Wave 3 | 0.059*** | [0.032, 0.086] |
| Wave 4 | 0.061*** | [0.030, 0.091] |
| **DMN-CON** |  |  |
| Wave 2 | 0.041*** | [0.017, 0.065] |
| Wave 3 | 0.042** | [0.015, 0.068] |
| Wave 4 | 0.042** | [0.011, 0.073] |
| **SN-CON** |  |  |
| Wave 2 | 0.035** | [0.012, 0.058] |
| Wave 3 | 0.043*** | [0.017, 0.069] |
| Wave 4 | 0.051*** | [0.021, 0.081] |
| **VAN-DAN** |  |  |
| Wave 2 | 0.043*** | [0.020, 0.067] |
| Wave 3 | 0.050*** | [0.024, 0.077] |
| Wave 4 | 0.058*** | [0.028, 0.088] |
| **VAN-CON** |  |  |
| Wave 2 | 0.047*** | [0.023, 0.070] |
| Wave 3 | 0.051*** | [0.024, 0.077] |
| Wave 4 | 0.055*** | [0.025, 0.085] |
| **DAN-DAN** |  |  |
| Wave 2 | -0.036** | [-0.060, -0.013] |
| Wave 3 | -0.041** | [-0.067, -0.014] |
| Wave 4 | -0.045** | [-0.075, -0.015] |

*Note.* Follow-up tests of associations between resting-state functional connectivity networks and p-factor scores at Waves 2 (one-year follow-up), 3 (two-year follow-up), and 4 (three-year follow-up) were conducted when relations with intercepts, but not slopes, were significant in the main analyses (see Table 3). Standardized estimates are shown. ***unadjusted *p*<0.001; *unadjusted *p*<0.05. DMN = default mode network; SN = salience network; VAN = ventral attention network; DAN = dorsal attention network; CON = cingulo-opercular network.

**Table S6*.*** *Exploratory Within- and Between Network Resting-State Functional Connectivity Relations with Intercept and Slope of P-Factor Scores Over Wave.*

| **Resting-State Functional Connectivity Networks** | **Intercept (Main Effect)** | | **Quadratic Slope (Interaction Effect)** | | **Linear Slope (Interaction Effect)** | |
| --- | --- | --- | --- | --- | --- | --- |
| *Exploratory Networks* | **β** | **95% CI** | **β** | **95% CI** | **β** | **95% CI** |
| DMN-AD | -0.010 | [-0.030, 0.011] | -0.002 | [-0.007, 0.003] | 0.011 | [-0.005, 0.028] |
| DMN-CA | 0.006 | [-0.014, 0.026] | 0.003 | [-0.002, 0.008] | -0.009 | [-0.025, 0.008] |
| DMN-N | 0.003 | [-0.019, 0.025] | -0.002 | [-0.008, 0.003] | 0.006 | [-0.010, 0.023] |
| DMN-RSPLTP | 0.002 | [-0.018, 0.022] | 0.002 | [-0.003, 0.007] | -0.003 | [-0.019, 0.014] |
| DMN-SMH | 0.013 | [-0.007, 0.033] | 0.000 | [-0.005, 0.005] | 0.003 | [-0.013, 0.019] |
| DMN-SMM | -0.008 | [-0.028, 0.012] | -0.001 | [-0.006, 0.004] | 0.009 | [-0.008, 0.025] |
| DMN-VS | 0.001 | [-0.020, 0.022] | 0.004 | [-0.001, 0.009] | -0.011 | [-0.028, 0.005] |
| FPN-AD | -0.003 | [-0.023, 0.017] | 0.000 | [-0.005, 0.006] | 0.006 | [-0.010, 0.023] |
| FPN-CA | 0.003 | [-0.017, 0.022] | 0.001 | [-0.004, 0.007] | -0.005 | [-0.022, 0.011] |
| FPN-N | 0.001 | [-0.020, 0.021] | 0.000 | [-0.005, 0.006] | -0.003 | [-0.020, 0.013] |
| FPN-RSPLTP | -0.004 | [-0.024, 0.017] | -0.002 | [-0.007, 0.003] | 0.013 | [-0.004, 0.029] |
| FPN-SMH | -0.007 | [-0.027, 0.014] | -0.002 | [-0.007, 0.004] | 0.019* | [0.003, 0.036] |
| FPN-SMM | 0.007 | [-0.013, 0.028] | 0.002 | [-0.004, 0.007] | 0.000 | [-0.016, 0.016] |
| FPN-VS | 0.001 | [-0.019, 0.022] | 0.003 | [-0.002, 0.008] | -0.009 | [-0.026, 0.007] |
| SN-AD | 0.022* | [0.001, 0.042] | 0.004 | [-0.001, 0.009] | -0.012 | [-0.029, 0.004] |
| SN-CA | 0.004 | [-0.016, 0.025] | 0.001 | [-0.004, 0.007] | -0.007 | [-0.024, 0.009] |
| SN-N | -0.008 | [-0.028, 0.012] | -0.002 | [-0.007, 0.003] | 0.005 | [-0.012, 0.021] |
| SN-RSPLTP | 0.004 | [-0.016, 0.025] | 0.001 | [-0.004, 0.006] | -0.002 | [-0.018, 0.015] |
| SN-SMH | 0.018 | [-0.002, 0.039] | 0.001 | [-0.005, 0.006] | 0.005 | [-0.012, 0.021] |
| SN-SMM | 0.009 | [-0.011, 0.029] | -0.003 | [-0.008, 0.002] | 0.007 | [-0.009, 0.023] |
| SN-VS | -0.014 | [-0.034, 0.006] | 0.000 | [-0.005, 0.005] | 0.003 | [-0.014, 0.019] |
| VAN-AD | 0.026* | [0.006, 0.047] | 0.003 | [-0.002, 0.008] | -0.014 | [-0.031, 0.002] |
| VAN-CA | -0.023* | [-0.043, -0.003] | -0.004 | [-0.009, 0.002] | 0.012 | [-0.005, 0.028] |
| VAN-N | -0.013 | [-0.033, 0.008] | -0.004 | [-0.009, 0.001] | 0.011 | [-0.005, 0.028] |
| VAN-RSPLTP | 0.004 | [-0.017, 0.024] | 0.000 | [-0.005, 0.006] | -0.005 | [-0.021, 0.012] |
| VAN-SMH | 0.015 | [-0.005, 0.035] | 0.002 | [-0.004, 0.007] | -0.002 | [-0.018, 0.015] |
| VAN-SMM | 0.011 | [-0.009, 0.031] | 0.003 | [-0.002, 0.008] | -0.009 | [-0.025, 0.007] |
| VAN-VS | -0.002 | [-0.023, 0.018] | 0.003 | [-0.002, 0.008] | -0.012 | [-0.028, 0.005] |
| DAN-AD | 0.018 | [-0.002, 0.038] | 0.007** | [0.002, 0.013] | -0.018* | [-0.034, -0.002] |
| DAN-CA | -0.014 | [-0.034, 0.006] | -0.005 | [-0.010, 0.000] | 0.021** | [0.005, 0.038] |
| DAN-N | 0.015 | [-0.006, 0.035] | 0.003 | [-0.002, 0.009] | -0.013 | [-0.029, 0.004] |
| DAN-RSPLTP | -0.001 | [-0.021, 0.019] | -0.004 | [-0.009, 0.001] | 0.011 | [-0.005, 0.027] |
| DAN-SMH | -0.009 | [-0.029, 0.010] | 0.002 | [-0.003, 0.008] | -0.005 | [-0.021, 0.012] |
| DAN-SMM | 0.027** | [0.007, 0.047] | 0.005 | [0.000, 0.010] | -0.014 | [-0.030, 0.003] |
| DAN-VS | -0.005 | [-0.025, 0.016] | 0.000 | [-0.005, 0.005] | 0.003 | [-0.013, 0.020] |
| CON-AD | 0.021* | [0.001, 0.042] | 0.000 | [-0.006, 0.005] | 0.003 | [-0.014, 0.019] |
| CON-CA | -0.023* | [-0.043, -0.003] | 0.001 | [-0.004, 0.007] | -0.002 | [-0.019, 0.014] |
| CON-N | 0.008 | [-0.013, 0.029] | 0.000 | [-0.005, 0.006] | -0.005 | [-0.021, 0.012] |
| CON-RSPLTP | 0.013 | [-0.008, 0.033] | 0.005 | [0.000, 0.010] | -0.023** | [-0.039, -0.007] |
| CON-SMH | 0.016 | [-0.005, 0.036] | -0.003 | [-0.008, 0.002] | 0.009 | [-0.007, 0.026] |
| CON-SMM | 0.022* | [0.002, 0.042] | -0.001 | [-0.006, 0.004] | -0.001 | [-0.018, 0.015] |
| CON-VS | -0.017 | [-0.037, 0.003] | 0.003 | [-0.003, 0.008] | -0.009 | [-0.025, 0.007] |
| AD-AD | -0.005 | [-0.025, 0.016] | -0.002 | [-0.008, 0.003] | 0.005 | [-0.012, 0.021] |
| AD-CA | 0.007 | [-0.013, 0.027] | -0.003 | [-0.008, 0.003] | 0.002 | [-0.015, 0.018] |
| AD-N | -0.021* | [-0.041, 0.000] | -0.001 | [-0.006, 0.004] | 0.006 | [-0.010, 0.023] |
| AD-RSPLTP | 0.009 | [-0.011, 0.030] | 0.006* | [0.001, 0.012] | -0.028** | [-0.045, -0.012] |
| AD-SMH | 0.000 | [-0.020, 0.020] | -0.001 | [-0.006, 0.004] | -0.003 | [-0.019, 0.014] |
| AD-SMM | 0.001 | [-0.019, 0.022] | 0.000 | [-0.005, 0.005] | -0.010 | [-0.026, 0.006] |
| AD-VS | 0.019 | [-0.001, 0.040] | 0.004 | [-0.001, 0.009] | -0.026** | [-0.042, -0.009] |
| CA-CA | -0.010 | [-0.030, 0.011] | 0.002 | [-0.003, 0.007] | -0.004 | [-0.020, 0.012] |
| CA-N | -0.010 | [-0.030, 0.010] | 0.000 | [-0.005, 0.006] | -0.007 | [-0.024, 0.009] |
| CA-RSPLTP | 0.009 | [-0.011, 0.029] | 0.000 | [-0.005, 0.005] | 0.002 | [-0.015, 0.018] |
| CA-SMH | 0.013 | [-0.007, 0.033] | 0.001 | [-0.004, 0.006] | -0.006 | [-0.022, 0.011] |
| CA-SMM | 0.002 | [-0.018, 0.022] | -0.003 | [-0.008, 0.002] | 0.005 | [-0.011, 0.021] |
| CA-VS | 0.019 | [-0.001, 0.039] | 0.000 | [-0.005, 0.006] | 0.000 | [-0.017, 0.016] |
| N-N | 0.008 | [-0.013, 0.030] | 0.002 | [-0.003, 0.007] | -0.011 | [-0.027, 0.005] |
| N-RSPLTP | -0.005 | [-0.025, 0.015] | 0.000 | [-0.005, 0.005] | 0.001 | [-0.016, 0.017] |
| N-SMH | -0.003 | [-0.023, 0.017] | -0.001 | [-0.006, 0.005] | 0.008 | [-0.008, 0.025] |
| N-SMM | -0.004 | [-0.024, 0.016] | 0.001 | [-0.005, 0.006] | 0.003 | [-0.014, 0.019] |
| N-VS | -0.010 | [-0.031, 0.011] | 0.003 | [-0.002, 0.008] | -0.009 | [-0.025, 0.008] |
| RSPLTP-RSPLTP | -0.013 | [-0.034, 0.008] | -0.003 | [-0.008, 0.002] | 0.011 | [-0.005, 0.027] |
| RSPLTP-SMH | 0.005 | [-0.015, 0.025] | 0.001 | [-0.004, 0.006] | -0.014 | [-0.031, 0.002] |
| RSPLTP-SMM | 0.012 | [-0.008, 0.032] | 0.004 | [-0.001, 0.010] | -0.022** | [-0.038, -0.005] |
| RSPLTP-VS | 0.005 | [-0.015, 0.025] | 0.002 | [-0.004, 0.007] | -0.003 | [-0.020, 0.013] |
| SMH-SMH | 0.005 | [-0.015, 0.026] | -0.003 | [-0.008, 0.002] | -0.006 | [-0.022, 0.011] |
| SMH-SMM | 0.020 | [-0.001, 0.040] | 0.003 | [-0.003, 0.008] | -0.019* | [-0.036, -0.003] |
| SMH-VS | 0.009 | [-0.011, 0.030] | 0.003 | [-0.002, 0.009] | -0.019* | [-0.036, -0.003] |
| SMM-SMM | -0.020 | [-0.040, 0.001] | -0.001 | [-0.006, 0.004] | -0.003 | [-0.019, 0.014] |
| SMM-VS | -0.002 | [-0.022, 0.018] | -0.001 | [-0.006, 0.004] | -0.004 | [-0.021, 0.012] |
| VS-VS | -0.003 | [-0.024, 0.019] | -0.003 | [-0.008, 0.002] | 0.013 | [-0.004, 0.029] |

*Note.* * unadjusted *p*<0.05; ** unadjusted *p*<0.01; *** unadjusted *p*<0.001; none of the estimates survived (*p*>0.05) FDR correction for 210 exploratory analyses; DMN = default mode network; FPN = frontoparietal network; SN = salience network; VAN = ventral attention network; DAN = dorsal attention network; CON = cingulo-opercular network; VS = visual network; AD = auditory network; CA = cingulo-parietal network; RSPLTP = retrospenial temporal network; SMM = sensorimotor mouth network; SMH = sensorimotor hand network; N = “none” network.

**Table S7.** *Sensitivity Analyses of* *Within- and Between Network Resting State Functional Connectivity Relations with the Intercept and Slope of P-Factor Scores Over Wave.*

| **Resting State Functional Connectivity Networks** | **Intercept (Main Effect)** | | **Slope (Interaction Effect)** | |
| --- | --- | --- | --- | --- |
|  | **β** | **95% CI** | **β** | **95% CI** |
| *Medication Covariate* |  |  |  |  |
| DMN-DMN | -0.026* | [-0.047, -0.005] | -0.003 | [-0.008, 0.002] |
| DMN-VAN | -0.029** | [-0.049, -0.009] | -0.008** | [-0.013, -0.003] |
| DMN-DAN | 0.055*** | [0.034, 0.076] | 0.002 | [-0.003, 0.007] |
| DMN-CON | 0.039*** | [0.019, 0.060] | 0.000 | [-0.005, 0.006] |
| SN-CON | 0.025* | [0.005, 0.045] | 0.008** | [0.003, 0.013] |
| VAN-DAN | 0.036*** | [0.015, 0.056] | 0.007** | [0.002, 0.012] |
| VAN-CON | 0.038*** | [0.018, 0.059] | 0.004 | [-0.001, 0.009] |
| DAN-DAN | -0.031** | [-0.051, -0.011] | -0.004 | [-0.009, 0.001] |
| *Excluded for High-Motion (FD<0.2mm)* | | | | |
| DMN-DMN | -0.032* | [-0.059, -0.005] | -0.006 | [-0.012, -0.001] |
| DMN-VAN | -0.028* | [-0.055, -0.002] | -0.011** | [-0.017, -0.004] |
| DMN-DAN | 0.060*** | [0.034, 0.087] | 0.009** | [0.002, 0.015] |
| DMN-CON | 0.035* | [0.008, 0.061] | 0.001 | [-0.005, 0.008] |
| SN-CON | 0.050*** | [0.025, 0.075] | 0.007* | [0.001, 0.014] |
| VAN-DAN | 0.030* | [0.004, 0.056] | 0.012*** | [0.005, 0.018] |
| VAN-CON | 0.058*** | [0.031, 0.083] | 0.006 | [-0.001, 0.012] |
| DAN-DAN | -0.035** | [-0.061, -0.010] | -0.007* | [-0.014, -0.001] |
| *Excluded for Completely Missing Follow-Up Data* | | | | |
| DMN-DMN | -0.033** | [-0.054, -0.011] | -0.003 | [-0.008, 0.002] |
| DMN-VAN | -0.031** | [-0.052, -0.010] | -0.008** | [-0.014, -0.003] |
| DMN-DAN | 0.059*** | [0.037, 0.080] | 0.002 | [-0.003, 0.007] |
| DMN-CON | 0.044*** | [0.022, 0.065] | 0.001 | [-0.005, 0.006] |
| SN-CON | 0.028** | [0.008, 0.049] | 0.008** | [0.003, 0.013] |
| VAN-DAN | 0.040*** | [0.019, 0.061] | 0.007** | [0.002, 0.013] |
| VAN-CON | 0.042*** | [0.021, 0.063] | 0.004 | [-0.001, 0.009] |
| DAN-DAN | -0.036*** | [-0.056, -0.015] | -0.004 | [-0.010, 0.001] |

*Note.* There were two associations that were significant in the low motion sample (i.e., FD<0.2mm), that we did not find in the primary analyses. Specifically, lower baseline within-DAN connectivity and higher DMN-DAN connectivity was positively associated with the slope of p-factor scores over wave. However, these associations should be interpreted with caution given that excluding participants with high motion can restrict variation on the p-factor. * unadjusted p<0.05; ** unadjusted p<0.01; *** unadjusted p<0.001; DMN = default mode network; SN = salience network; VAN = ventral attention network; DAN = dorsal attention network; CON = cingulo-opercular network.

**Figure Legends**

**Figure S1.** *Flowchart of Participant Inclusion/Exclusion Criteria.*

Note: Out of the 11,876 ABCD baseline participants, 9,374 passed fMRI quality control (QC) information as recommended by the ABCD Data Analysis and Informatics Resource Center (DAIRC). Out of those 9,374 participants, there were 26 participants excluded due to a small number of participants in that site. From the remaining 9,348 participants, 4 were excluded due to missing baseline Child Behavior Checklist (CBCL) data. The total baseline sample included 9,344 participants. Out of those 9,344 participants, 8,840 participants had available CBCL data at Wave 2, 8,618 had available data at Wave 3, and 8,034 had available data at Wave 4.

**Figure S1.**
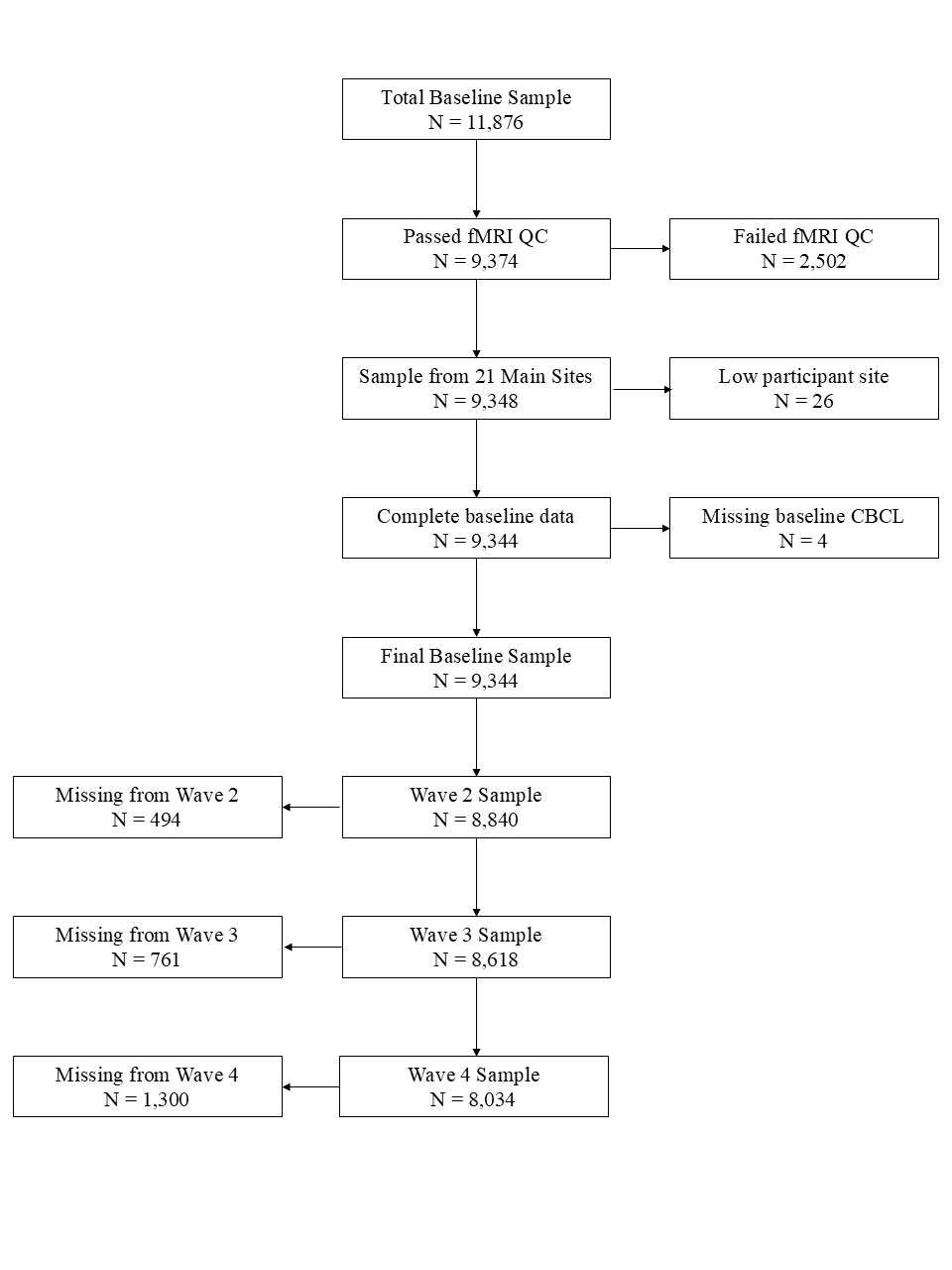

Supplement: Supplementary file 1 — Supporting Information S1 [file JCV2-9999-e70135-s001.docx]
